# Nicotine-mediated effects in neuronal and mouse models of synucleinopathy

**DOI:** 10.3389/fnins.2023.1239009

**Published:** 2023-08-31

**Authors:** Mohamed Bilal Fares, Omar Alijevic, Stephanie Johne, Cassia Overk, Makoto Hashimoto, Athanasios Kondylis, Anthony Adame, Remi Dulize, Dariusz Peric, Catherine Nury, James Battey, Emmanuel Guedj, Nicolas Sierro, Damian Mc Hugh, Edward Rockenstein, Changyoun Kim, Robert A. Rissman, Julia Hoeng, Manuel C. Peitsch, Eliezer Masliah, Carole Mathis

**Affiliations:** ^1^PMI R&D, Philip Morris Products S.A., Neuchâtel, Switzerland; ^2^Department of Neurosciences, University of California, San Diego, San Diego, CA, United States

**Keywords:** synucleinopathy, induced pluripotent stem cell (iPSC), transgenic mice, nicotine, nicotinic acetylcholine receptors (nAChR), neuroprotection

## Abstract

**Introduction:**

Alpha-synuclein (α-Syn) aggregation, transmission, and contribution to neurotoxicity represent central mechanisms underlying Parkinson’s disease. The plant alkaloid “nicotine” was reported to attenuate α-Syn aggregation in different models, but its precise mode of action remains unclear.

**Methods:**

In this study, we investigated the effect of 2-week chronic nicotine treatment on α-Syn aggregation, neuroinflammation, neurodegeneration, and motor deficits in D-line α-Syn transgenic mice. We also established a novel humanized neuronal model of α-Syn aggregation and toxicity based on treatment of dopaminergic neurons derived from human induced pluripotent stem cells (iPSC) with α-Syn preformed fibrils (PFF) and applied this model to investigate the effects of nicotine and other compounds and their modes of action.

**Results and discussion:**

Overall, our results showed that nicotine attenuated α-Syn-provoked neuropathology in both models. Moreover, when investigating the role of nicotinic acetylcholine receptor (nAChR) signaling in nicotine’s neuroprotective effects in iPSC-derived dopaminergic neurons, we observed that while α4-specific antagonists reduced the nicotine-induced calcium response, α4 agonists (e.g., AZD1446 and anatabine) mediated similar neuroprotective responses against α-Syn PFF-provoked neurodegeneration. Our results show that nicotine attenuates α-Syn-provoked neuropathology *in vivo* and in a humanized neuronal model of synucleinopathy and that activation of α4β2 nicotinic receptors might mediate these neuroprotective effects.

## Introduction

Alpha-synuclein (α-Syn) is a neuronal protein implicated in neuroplasticity and vesicle trafficking. Aggregated, fragmented, and post-transcriptionally modified α-Syn proteoforms are key components of Lewy bodies and Lewy neurites, which are pathological hallmarks of Parkinson’s disease (PD) and other synucleinopathies (Fares et al., 2021). Under diseased states, this conformationally plastic protein misfolds and undergoes a series of multimerization/oligomerization events, generating beta-sheet aggregates that subsequently grow and adopt fibrillar morphology. Increasing evidence from animal and cellular models suggests that different α-Syn aggregates and fibrillar fragments might spread among neurons and non-neuronal cells, perpetuating Lewy body pathology through a prion-like mechanism and triggering neuroinflammation (Lim et al., 2016). Accumulation of these aggregates has been also linked to the failure of protein-degradation mechanisms, as well as apoptosis in both cellular and animal models of PD (Desplats et al., 2009). Studies have shown that α-Syn aggregates can provoke dopaminergic toxicity via multiple interrelated processes (Volpicelli-Daley et al., 2011; Wang et al., 2019), including neuroinflammatory responses, impairment of mitochondrial activity, synaptic dysfunction, and oxidative/nitrative stress.

In the quest for molecules that could alleviate the toxic impact of α-Syn aggregates, alkaloids have been investigated because they offer myriad structural arrangements that can modulate molecular targets exhibiting different conformations. Notably, some alkaloids have been reported to exhibit protective effects in different models of synucleinopathy, including salsoline, an alkaloid found in the Chenopodiaceae plant family; piperine, an alkaloid produced by long and black pepper (Hussain et al., 2018); and isorhynchophylline, an alkaloid extracted from *Uncaria rhynchophylla* (Lu et al., 2012). Nicotine—another plant alkaloid derived from tobacco, eggplant, and tomatoes—was demonstrated to attenuate α-Syn aggregation kinetics in cell-free systems, as well as in a yeast model of α-Syn aggregation (Ono K. et al., 2007; Hong et al., 2009; Kardani et al., 2017). Interest in assessing nicotine’s potential neuroprotective effects against PD and other synucleinopathies has been historically fueled by numerous epidemiological studies that have consistently demonstrated a two-fold risk reduction of developing PD in subjects with a history of tobacco use (Ritz et al., 2007; Veljkovic et al., 2018). As nicotine is the major alkaloid found in tobacco, potential beneficial effects of nicotine *per se* have been extensively investigated in different cellular and animal models of PD (Quik and Jeyarasasingam, 2000; Riveles et al., 2008; Xie et al., 2014; Srinivasan et al., 2016; Kardani et al., 2017). Importantly, a multitude of these studies in the past three decades have reported beneficial effects of nicotine in different preclinical models of disease, but effects in several PD clinical trials have yielded conflicting results (Fagerstrom et al., 1994; Clemens et al., 1995; Kelton et al., 2000; Vieregge et al., 2001; Lemay et al., 2004; Villafane et al., 2007; Itti et al., 2009; Holmes et al., 2011). However, the effort to explore the therapeutic potential of nicotine is still ongoing with, for example, the Memory Improvement through Nicotine Delivery (MIND) study assessing nicotine impact on cognition (in partnership with the National Institutes of Health (NIH), Alzheimer’s Drug Discovery Foundation, Vanderbilt University & University of Southern California) or on Parkinson’s disease-related dyskinesia, suggesting that interest in assessing the therapeutic potential of nicotine is still present (Newhouse, 2019; Alhowail, 2021). The mechanism involved in nicotine-mediated neuroprotective effects also remains to be clarified, but given its known ability to bind and activate nicotinic acetylcholine receptors (nAChR), it has been proposed that activation of these receptors may be involved in this process, together with potential direct effects on α-Syn aggregation (Veljkovic et al., 2018).

In this study, we investigated whether nicotine modulates the toxic impact of α-Syn aggregates using two different models of synucleinopathy: (1) an *in vivo* mouse model overexpressing α-Syn, and (2) a humanized neuronal model based on the treatment of induced pluripotent stem cells (iPSC) with α-Syn aggregates. The *in vivo* studies employed D-Line transgenic (tg) mice overexpressing human α-Syn under the platelet-derived growth factor-β (PDGFβ) promoter (PDGFβ α-Syn), which we have developed and extensively utilized to investigate the interplay between α-Syn aggregation and neuropathological and behavioral deficits (Masliah et al., 2005, 2011; Lee et al., 2010; Amschl et al., 2013; Kim et al., 2018). Following 2-week chronic treatment with nicotine, α-Syn tg mice were tested for potential behavioral improvement by the rotarod test, and relevant brain tissues were assessed by immunohistochemical analysis of neurodegeneration and neuroinflammation markers. In addition, the impact of nicotine on α-Syn aggregation was evaluated by immunohistochemistry using antibodies targeting total and pS129 α-Syn. Using this *in vivo* model, we detected significant reduction in α-Syn aggregates following nicotine treatment, which was concomitant with attenuated neurodegeneration, lower levels of neuroinflammatory markers, and improved motor function.

To investigate the effect of nicotine on dopaminergic neurons and the involvement of the nicotinic signaling pathway in mediating neuroprotective effects, we developed a humanized neuronal model of synucleinopathy by differentiating human iPSCs into dopaminergic neurons. Interestingly, whereas lentiviral-mediated α-Syn overexpression provoked minimal pathology in this model, treatment with α-Syn preformed fibrils (α-Syn PFF) consistently triggered toxic events in a time- and dose-dependent manner. Importantly, nicotine pretreatment significantly attenuated the toxic effects of α-Syn PFF, without affecting their uptake and internalization by neurons. After validating nAChR expression and functionality in our dopaminergic model, we investigated whether nAChR activation could underlie the observed nicotine-mediated neuroprotection, and if other related tobacco alkaloids could exhibit similar effects. Notably, our results show that nicotine and another related alkaloid, anatabine, exhibit similar neuroprotective effects against α-Syn PFF-provoked neurodegeneration in iPSC-derived dopaminergic neurons, and suggest that α4β2 nicotinic acetylcholine signaling might be involved in mediating these effects.

## Materials and methods

### Animal model and treatment

For *in vivo* experiments, we used 12-month-old (male and female) mice overexpressing human α-Syn under the PDGFβ promoter (PDGFβ α-Syn) which were either high (Line D) or medium expressers of α-Syn (Masliah et al., 2000), along with age-matched (male and female) non-tg mouse littermates. A total of 40 (*n* = 20 tg and *n* = 20 non-tg) age-matched and sex-balanced mice were used to ensure sufficient power of statistical analyses. In each group, 10 non-tg and 10 tg mice, which were 12 months old at the start of the treatment, were injected with saline control or nicotine (0.1 mg/kg intraperitoneal [IP]) twice daily for 2 weeks.

### Ethics statement

All *in vivo* experiments were conducted in compliance with the ARRIVE guidelines, in accordance with the NIH guidelines for animal use, and according to ethical policies and procedures approved by the Institutional Animal Care and Use Committee of the University of California San Diego (UCSD; approval no. S02221). All *in vivo* experiments were randomized and computed for power.

### Rotarod test

Measurements using the ROTOR-ROD™ system (San Diego Instruments, San Diego, CA, USA) were carried out over 2 consecutive days as we previously reported (Masliah et al., 2000, 2011). The first day represented “training day,” where mice were conditioned over five trials at different speeds: (10 and 20 rpm for the first two runs and 40 rpm for the last three). The actual test runs were conducted on the second day over seven trials at 40 rpm (acceleration 0–40 rpm over 240 s). Fall latency was recorded by assessing the amount of time spent by the mice on the rod, and the average results of all tested mice at each trial were calculated and presented.

### Immunohistochemical and image analyses

Immunohistochemical analysis was performed by incubating serial sections of vibratome-sliced brains with appropriate primary antibodies (Supplementary Table 1) overnight at 4°C, as we previously reported (Mandler et al., 2019). The sections were then exposed to biotinylated secondary antibodies (1,100; Vector Laboratories, Inc., Burlingame, CA, USA) and avidin–biotin complex (1,200; ABC Elite, Vector Laboratories, Inc.) before being developed with diaminobenzidine (DAB). The brain slices developed with DAB were then imaged using aB50XS Olympus microscope (Olympus, Tokyo, Japan), and images were quantified using ImageJ software (NIH, Bethesda, MD, USA) to determine signal intensities (Ubhi et al., 2010, 2012; Bae et al., 2012). To count numbers of NeuN-positive cells, brain slices were imaged using an Olympus BX51 microscope operating the StereoInvestigator 8.21.1 software (MBF Bioscience, Williston, VT, USA) and using a 100× 1.4 PlanApo oil-immersion objective as previously reported (Overk et al., 2009). The sizes of the grids of the hippocampal cornu ammonis 3 and 1 (CA3 and CA1) pyramidal layers were 150 μm × 150 μm and 300 μm × 300 μm, respectively, and the counting frames were 30 μm × 30 μm and 50 μm × 50 μm, respectively.

### iPSC culture and maintenance

The Gibco™ Human Episomal iPSC Line A18945 (Thermo Fisher Scientific, Waltham, MA, USA) was used throughout this study. This line was generated by transfecting cord blood cells with three plasmids encoding seven factors (SOKMNLT; sex-determining region Y-box 2 [SOX2], OCT4 [POU5F1], Krüppel-like factor 4 [KLF4], MYC, NANOG, LIN28A, and SV40L T antigen). The system is Epstein–Barr virus nuclear antigen-based (episomal) and therefore involves no genomic integration. iPSCs were cultured on vitronectin-coated plates in Essential 8™ Flex medium, with manual passaging every 4–5 days with Accutase® (Thermo Fisher Scientific) at a 1:6 ratio.

### iPSC differentiation into midbrain dopaminergic neurons

Differentiation of iPSCs into dopaminergic neurons was performed by using the Gibco™ PSC Dopaminergic Neuron Differentiation kit (Thermo Fisher Scientific), as outlined in Supplementary Figure 2A and according to the manufacturer’s instructions. iPSCs were first plated on vitronectin-coated dishes at 1.0 × 10^4^ viable cells/cm^2^ in Essential 8™ Flex medium. The following day, the cells were specified into mid-brain-specified Floor Plate progenitor (FPp) cells by switching their medium to Floor Plate Specification medium for 10 days. Over the next 7 days, the FPp cells were expanded in Floor Plate Cell Expansion medium in laminin-coated plates into FPp1 cells and subsequently into FPp2 cells, which were then stored in liquid nitrogen or directly expanded in suspension for 7 days to form spheres. Finally, the spheres were triturated with Accutase® (Thermo Fisher Scientific), plated at 0.2 × 10^6^ cells/cm^2^, and differentiated into mature dopaminergic neurons in dopaminergic neuron maturation medium (M1; DMEM/F-12 medium with GlutaMAX™ [Thermo Fisher Scientific] and maturation additive [Thermo Fisher Scientific]) in poly-D-lysine/laminin double-coated dishes. Neurons were plated on clear-bottom 96-well plates for immunocytochemical analysis and calcium imaging/recording, in 6-well plates for biochemical analysis and automated patch clamp recordings, and in 96-well E-Plates (ACEA Biosciences, San Diego, CA, USA) for monitoring effects on cell adhesion in real-time using the xCELLigence™ system (ACEA Biosciences). The xCELLigence™ readout is based on measuring electron flow between gold microelectrodes placed at the bottom of proprietary E-plates, and cell adhesion to the plate surface impedes this electrical flow, enabling the measurement of cell index over time. To determine cell viability, the total ATP content of cultures was analyzed using the ATP assay kit from Promega as per standard procedure detailed by the manufacturer (CellTiter-Glo® Luminescent Cell Viability Assay, Promega, Madison, WI, USA). In all cases, half of the neuronal medium of cultured neurons was replaced with fresh medium every 2–3 days over a period of 7 days, and mature neurons were then maintained in dopaminergic neuron maturation medium (M2; neurobasal medium with GlutaMAX™ [Thermo Fisher Scientific], B27 [Thermo Fisher Scientific], and maturation supplement [Thermo Fisher Scientific]), with subsequent half-volume medium changes every 2–3 days. At each passaging, the cells were treated with ROCK inhibitor (Y27632; Sigma, St. Louis, MO, USA) to enhance survival rates. The entire workflow takes approximately 35–42 days, and the cultures were incubated in humidified incubators at 37°C and 5% CO2 throughout all experiments.

### Lentiviral-mediated α-Syn transduction

Titrated lentiviruses encoding human α-Syn, an empty vector (as negative control), or green fluorescent protein (GFP) were acquired from AMSBIO (AMS Biotechnology Ltd., Massagno, Switzerland). To induce overexpression, half of the neuronal culture medium was stored at 4°C, and the other half was transferred to a test tube to which aliquots of premade viral stocks thawed at room temperature were also added to achieve the required multiplicity of infection (MOI). Cells were then treated with the mix and maintained in a 37°C/CO2 incubator over 24 h. The previously refrigerated medium was warmed at 37°C and added to the cells, which were then maintained for the desired duration in a 37°C/CO2 incubator until further analysis or treatment.

### α-Syn monomer and PFF characterization

Human α-Syn monomers (M) and PFF were purchased from Proteos Inc. (Kalamazoo, MI, USA). The PFF were briefly sonicated with the Sonifier W450-D (Branson Ultrasonic Corp., Danbury, CT, USA) (10% amplitude, 3 pulses of 3 s), aliquoted, and stored at −80°C. The α-Syn M were analyzed by electrospray ionization-mass spectrometry (ESI-MS) by using the QExactive Plus system (Thermo Fisher Scientific) to determine the exact mass. The amyloid contents of α-Syn PFF (pre- and post-sonication) were assessed by measuring their binding to thioflavin-T (ThT; Sigma), sedimentation, and their length by transmission electron microscopy (TEM). To measure ThT binding, the proteins were mixed with 10 μM ThT in 50 mM glycine (pH 8.5) in black 96-well plates (Merck, Darmstadt, Germany), before reading fluorescence (excitation at 450 nm; emission at 485 nm) using a FLUOstar® plate reader (BMG LabTech, Ortenberg, Germany). To assess monomer release into the soluble fraction, proteins were centrifuged at 20,000 × g (10 min, 4°C), and aliquots of the supernatant fraction were run on NuPAGE™ Novex 4–12% (vol/vol) Bis-Tris gels (Thermo Fisher Scientific), which were then visualized with SYPRO® Ruby stain (Thermo Fisher Scientific). To validate the fibrillar ultrastructure of the PFF, samples were incubated for 60 s on Formvar/carbon-coated 200-mesh copper grids (Electron Microscopy Sciences, Hatfield, PA, USA), before being dried with filter paper, washed two times with water, and then incubated for 30 s with uranyl acetate 2% (w/v) (Electron Microscopy Sciences). Samples were finally dried using blotting paper and vacuum suction, and imaged by Microtrace LLC (Elgin, IL, USA) using a Philips CM12 system (Amsterdam, Netherlands).

### Immunocytochemical analysis of iPSC-derived neurons

As previously described (Fares et al., 2016), following a wash with phosphate-buffered saline (PBS), cells were fixed with 4% (v/v) paraformaldehyde (Thermo Fischer Scientific) in PBS. The presence of apoptotic neurons was investigated by performing the Click-iT® TUNEL assay (Thermo Fischer Scientific) according to the manufacturer’s instructions. The fixed neurons were first permeabilized upon incubation with 0.25% Triton™ X-100 over a period of 20 min at room temperature, washed twice with water, and then incubated with the TdT reaction cocktail over 1 h at 37°C. After two quick washes with 3% bovine serum albumin (BSA) in PBS, the neurons were treated with the Click-iT® reaction buffer over 30 min at room temperature, and then blocked for 1 h at room temperature in blocking buffer (3% [w/v] BSA, 0.02% saponin in PBS). The cells were subsequently incubated overnight at 4°C in blocking buffer containing appropriate primary antibodies (Supplementary Table 1). Following three washes with PBS, the cells were incubated for 1 h at room temperature in blocking buffer containing appropriate fluorescent-labeled secondary antibodies (Thermo Fischer Scientific) and the nuclear counterstain Hoechst 33342 (1:1000; Thermo Fischer Scientific). Cells were then washed three times with PBS, once with double-distilled water, and then imaged by confocal microscopy (Leica, Wetzlar, Germany) or by using the CellInsight™ CX7 high-content screening imaging platform (Thermo Fisher Scientific). In the latter case, nine fields per well were imaged by using a 10x wide-field objective, and fluorescence intensities were quantified by using the high-content screening Studio Cell Analysis Software (Thermo Fisher Scientific). FIJI open-source software (Schindelin et al., 2012) was also used for image quantification.

### Biochemical analysis of iPSC-derived neurons

Cells were treated with ice-cold lysis buffer (150 mM NaCl, 20 mM Trizma® base, 1 mM EDTA, 0.25% Nonidet™ P-40, and 0.25% Triton™ X-100; pH 7.4) containing phosphatase and protease inhibitors (Halt™ Protease and Phosphatase Inhibitor Cocktail, Thermo Fisher Scientific). After scraping and 20 min incubation on ice, lysates were centrifuged for 20 min at 14,000 × g (4°C), resulting in supernatants that comprise nonionic detergent-soluble fractions. The pellets were resuspended in lysis buffer containing 5% (w/v) SDS, before being sonicated for 15 s at 60% amplitude (3 s on/off) to obtain nonionic detergent-insoluble fractions. For each soluble and insoluble fraction, protein content was determined with the Pierce™ BCA protein assay kit (Thermo Fisher Scientific), and then proteins were resolved on Novex 4–12% (v/v) Bis-Tris NuPAGE™ gels (Thermo Fisher Scientific) before being blotted using the iBlot™ Transfer Cell (Invitrogen, Carlsbad, CA, USA) onto nitrocellulose membranes. Membranes were then blocked over 1 h at room temperature by incubating with Starting Block blocking buffer (Thermo Fisher Scientific), and then probed with appropriate primary antibodies overnight at 4°C (Supplementary Table 1). The membrane was then washed three times with PBST (PBS, 0.01% (v/v) and Tween®-20 [Thermo Fisher Scientific]), treated for 1 h with fluorescent-labeled secondary antibodies (Li-COR Biosciences, Lincoln, NE, USA), washed three times with PBST, and then scanned using an Odyssey® scanner (Li-COR Biosciences).

### DNA and RNA extraction and sequencing

DNA was isolated from samples by using the AllPrep DNA/RNA/Protein Mini kit from Qiagen (Hilden, Germany). DNA sequencing libraries were prepared by using the Celero DNA-seq library preparation kit (Tecan Genomics, Männedorf, Switzerland) and sequenced as 101-bp paired-end reads on an Illumina HiSeq® 2,500 sequencer and as 151-bp paired-end reads on an Illumina HiSeq® 4,000 sequencer (Illumina Inc., San Diego, CA, USA). The reads were cleaned of adapters and trimmed to a maximum length of 150 bases by using the bbduk tool version 37.99 (Bushnell, 2018). They were subsequently tagged using the GATK tool kit (DePristo et al., 2011) and aligned to the human genome (hg38, Ensembl release 78) by using the BWA-MEM algorithm version 0.7.17 (Li, 2013). Duplicates were removed from the alignment file GATK’s “MarkDuplicates.” The aligned and filtered reads were passed to the software Freebayes version 1.2.0 (Garrison and Marth, 2012) for variant calling. The resulting VCF file was filtered for a minimum quality score cutoff of QUAL>10, and the variants were annotated by using CAVA version 1.2.3 (Munz et al., 2015). The subset of mutations of direct relevance to the project were extracted from the VCF file by text matching.

RNA was isolated from the samples on a QIAcube by using the RNeasy Mini kit (Qiagen). RNA sequencing libraries were prepared by using the Universal Plus mRNA-Seq with NuQuant library preparation kit (Tecan Genomics) and sequenced as 150-bp paired-end reads on an Illumina HiSeq® 4,000 sequencer. The fastp tool was used to quality-check the raw sequencing reads and remove adapters and low-quality bases. The cleaned reads were then mapped to the Ensembl 96 human genome assembly, and the number of mapped reads per genomic feature was counted by using the STAR aligner (version 2.7.1a). Principal component analysis (PCA), differential gene expression, and Gene Ontology (GO) term enrichment were calculated using the PCATools (version 1.0.0), deseq2 (version 1.22.1), and ClusterProfiler (version 3.10.1) R packages, respectively.

Sequencing data are available at the National Center for Biotechnology Information Short Read Archive under accession PRJNA613205.

### Alkaloid preparation and exposure to iPSC-derived neurons

The tobacco compounds listed in Supplementary Table 2 were solubilized in cell culture medium to generate stock solutions of 1 mM concentration. Dose–response assessments of non-specific compound toxicity were performed over 1 week at concentrations ranging from 50 to 1,000 nM. Once the optimal treatment concentrations were determined, four different doses were tested to assess the potential neuroprotective effects of each of compound in the iPSC-derived neuronal model.

### Automated patch-clamp electrophysiology

An automated patch-clamp system (Patchliner Octo®, Nanion Technologies, Münich, Germany) was utilized as per standard procedure detailed by the manufacturer and as described previously (Haythornthwaite et al., 2012). Up to eight cells were measured simultaneously using the Patchliner Octo®, which is equipped with two EPC-10 Quadro patch-clamp amplifiers (HEKA Elektronik, Reutlingen, Germany). The external solution contained 4 mM KCl (Sigma), 140 mM NaCl (Sigma), 2 mM CaCl_2_ (Sigma), 1 mM MgCl_2_ (Sigma), 10 mM HEPES (Sigma), and 5 mM glucose (Sigma), at pH 7.4. The internal solution (60 mM KF, 50 mM KCl, 20 mM EGTA, 10 mM NaCl, 10 mM HEPES, and 1 mM MgCl_2_; Nanion) was supplemented with 3 mM MgCl_2_ (Sigma), 0.2 mM GTP (Sigma), and 3 mM ATP (Sigma), adjusted to pH 7.2. A seal-enhancer solution (3 mM KCl, 80 mM NaCl, 35 mM CaCl_2_, 10 mM MgCl_2_, and 10 mM HEPES [pH 7.4]; Nanion) was used and subsequently changed into external solution upon the establishment of the whole-cell configuration. Patch-ControlHT software (Nanion Technologies, version 12.0.1f9) and Patchmaster software (HEKA Elektronik, version 2×90.4 beta) were utilized for data acquisition. Data were recorded at a sampling rate of 20 kHz (at room temperature) and filtered at 5 kHz.

### Measurement of calcium changes using FLIPR Calcium 6

The FlexStation 3® and Calcium 6 Assay Kit were used in accordance with the manufacturer’s instructions (Molecular Devices, Sunnyvale, CA, USA). iPSC-derived dopaminergic neurons were plated on 96-well plates and maintained for 2 h at 37°C with 50 μL Calcium 6 dye (Molecular Devices) in a HEPES–HBSS (HBPS) solution (Thermo Fisher Scientific) containing 5.3 mM KCl, 138 mM NaCl, 1.2 mM CaCl_2_, 0.9 mM MgCl_2_, 5.5 mM glucose, and 10 mM HEPES (pH 7.4). During the recording (at a sampling rate of 1.3 s), neurotransmitter application was preceded by a 30-s baseline period and followed by addition of 30 mM KCl. Fluorescent changes were displayed by using SoftMax® Pro software (version 7, Molecular Devices).

### Measurement of calcium changes by single-cell imaging (Fura2)

iPSC-derived dopaminergic neurons were incubated with 3 μM Fura2-AM (Sigma) for 20 min at 37°C in HBPS solution in coated 96-well plates. The experiments were performed at room temperature. Images were recorded with a Hamamatsu camera (Hamamatsu Photonics K.K., Hamamatsu City, Japan) using an inverted fluorescent microscope (Eclipse Ti, Nikon, Tokyo, Japan) and analyzed with the NIS Element software (version 4.6, Nikon).

### Compounds used for functional analysis

All compounds were purchased from commercial sources and are listed in Supplementary Table 3. Stock solutions of the test compounds were prepared as 10–100 mM solutions in water or dimethyl sulfoxide (DMSO). All chemicals were stored at −20°C and prepared freshly at final concentrations in external recording solutions for patch-clamp and imaging recordings. Compounds dissolved from DMSO stock solutions contained a final concentration of 0.1% DMSO.

### Statistical analysis

Data are shown as mean ± standard deviation or ± standard error of the mean (as indicated in respective figure legends). Statistical significance was determined by calculating *p*-values using two-tailed Mann–Whitney U tests for two-group comparisons, one-way analysis of variance (ANOVA) followed by Dunnet’s post-hoc tests for multigroup comparisons to a single control, or Tukey–Kramer or Fisher post-hoc analysis for other multigroup comparisons. In all cases, statistical analyses were performed using JMP 12 software (SAS Institute Inc., Cary, NC, USA). Behavioral data were analyzed by two-way ANOVA. For RTCA plots, data points were expressed relative to time 0 (i.e., the time the treatment was applied), and effects were investigated at fixed time points following treatment. Experimental replicates from three independent experiments (*N* = 3) were generated, each including three technical replicates (*n* = 3). Statistical tests were done by means of two-tailed Student tests (*t*-tests). In all cases, *p*-values below 0.05 were considered significant, and no data exclusions were performed. Moreover, investigators were not blinded to group allocation, but a separate team of statisticians handled the data and performed all statistical analyses.

## Results

### Nicotine treatment reduces the accumulation of pathogenic α-Syn in tg mice

To investigate whether nicotine could exhibit neuroprotective effects against α-Syn-provoked pathology *in vivo,* we utilized the D-Line α-Syn tg model that we developed (Amschl et al., 2013), which accumulates α-Syn aggregates in the neocortex and limbic system, concomitant with axonal pathology, neuroinflammation, neurodegeneration, and behavioral deficits (Lee et al., 2010; Amschl et al., 2013). Moreover, we previously applied this model in several proof-of-concept studies that provided important preclinical data and informed the development of some of the antibody-based therapies targeting α-Syn that have advanced to clinical trials in patients with PD (Masliah et al., 2005, 2011; Kim et al., 2018). To this end, 12-month-old mice overexpressing human α-Syn (and non-tg controls) were treated with nicotine (0.1 mg/kg) or saline via IP injection twice daily for 2 weeks. The selection of this dose was based on a previous study that reported a neuroprotective effect of chronic nicotine treatment at low doses (0.75 and 1.5 mg/kg) against 6-hydroxydopamine (6-OHDA)-induced nigrostriatal degeneration (Ryan et al., 2001). To put this dose in the context of human nicotine exposure, an average of 1–1.5 mg of nicotine is absorbed systematically during smoking, which corresponds for an adult of 70–80 kg of body weight to a dose of 0.014–0.019 mg nicotine/kg (Benowitz et al., 2009).

**Figure 1:**
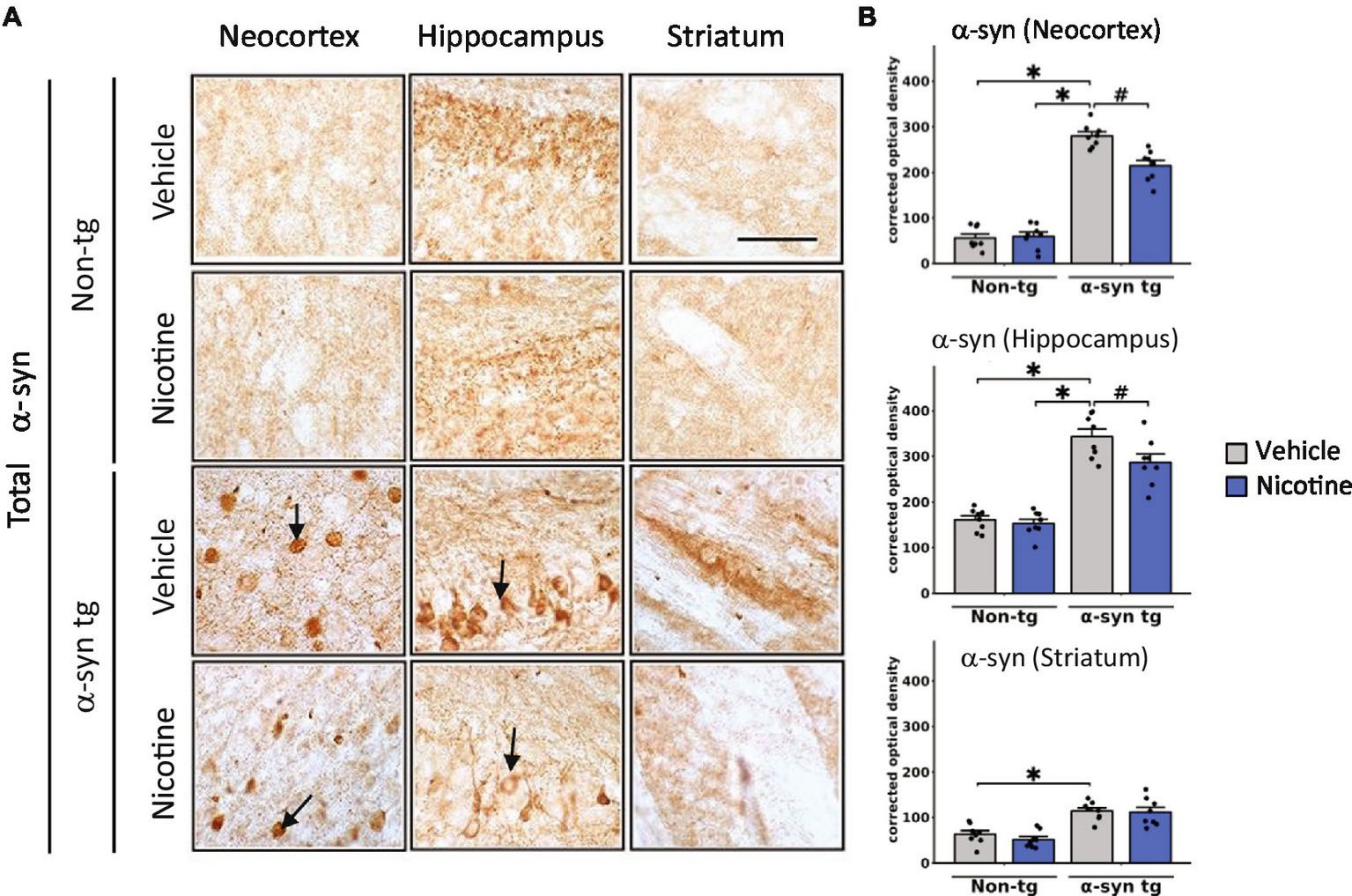
Immunohistological evaluation of the effect of nicotine treatment on α-Syn aggregation in transgenic mice. Twelve-month-old (male and female) mice overexpressing human α-syn under the PDGFβ promoter (PDGFβ α-syn) and non-tg mice were treated with nicotine (0.1 mg/kg IP) or saline (=vehicle) twice daily for 2 weeks. Brain sections from these mice were analyzed immunohistochemically for α-Syn immunoreactivity by using the Syn-1 antibody **(A,B)**. **(A)** Representative photomicrographs and **(B)** quantitative comparisons of α-Syn immunoreactivity in the neocortex, hippocampus, and striatum are shown. Relative to saline-treated non-tg mice, saline-treated α-Syn tg mice showed a significant increase in the number of α-Syn inclusions in the neocortex and hippocampus (see arrows), as well as greater neuropil immunoreactivity in the striatum. Nicotine treatment significantly reduced α-Syn inclusion formation in the neocortex and hippocampus but had no effect on α-Syn immunoreactivity in the neuropil of the striatum. *N* = 8 mice/group. Scale bar for all subpanels = 50 μm. ^♯^*p* < 0.05, *p < 0.0001. Dots represent individual values and bars denote the mean ± standard error of the mean.

Immunohistochemical studies were first performed to evaluate effects on α-Syn inclusions (Figure 1A). Consistent with previous findings, saline-treated α-Syn tg mice demonstrated α-Syn inclusions in the neocortex and hippocampus and neuropil in the striatum. Strikingly, nicotine-treated α-Syn tg mice showed significantly reduced formation of α-Syn inclusions in the neocortex and hippocampus relative to saline-treated α-Syn tg mice; however, there was no significant effect of nicotine treatment in the striatum of the α-Syn tg mice relative to saline-treated α-Syn tg mice. Similarly, pathological α-Syn accumulation into hyperphosphorylated inclusions at S129 (pS129) was significantly increased in the neocortex, hippocampus, and striatum of saline-treated α-Syn tg mice relative to saline-treated non-tg mice, and nicotine treatment significantly reduced pS129 α-Syn immuno reactivity in these brain regions relative to saline treatment (Supplementary Figures 1A,B).

### Nicotine treatment reduces neuroinflammation, neurodegeneration, and motor deficits in α-Syn tg mice

**Figure 2:**
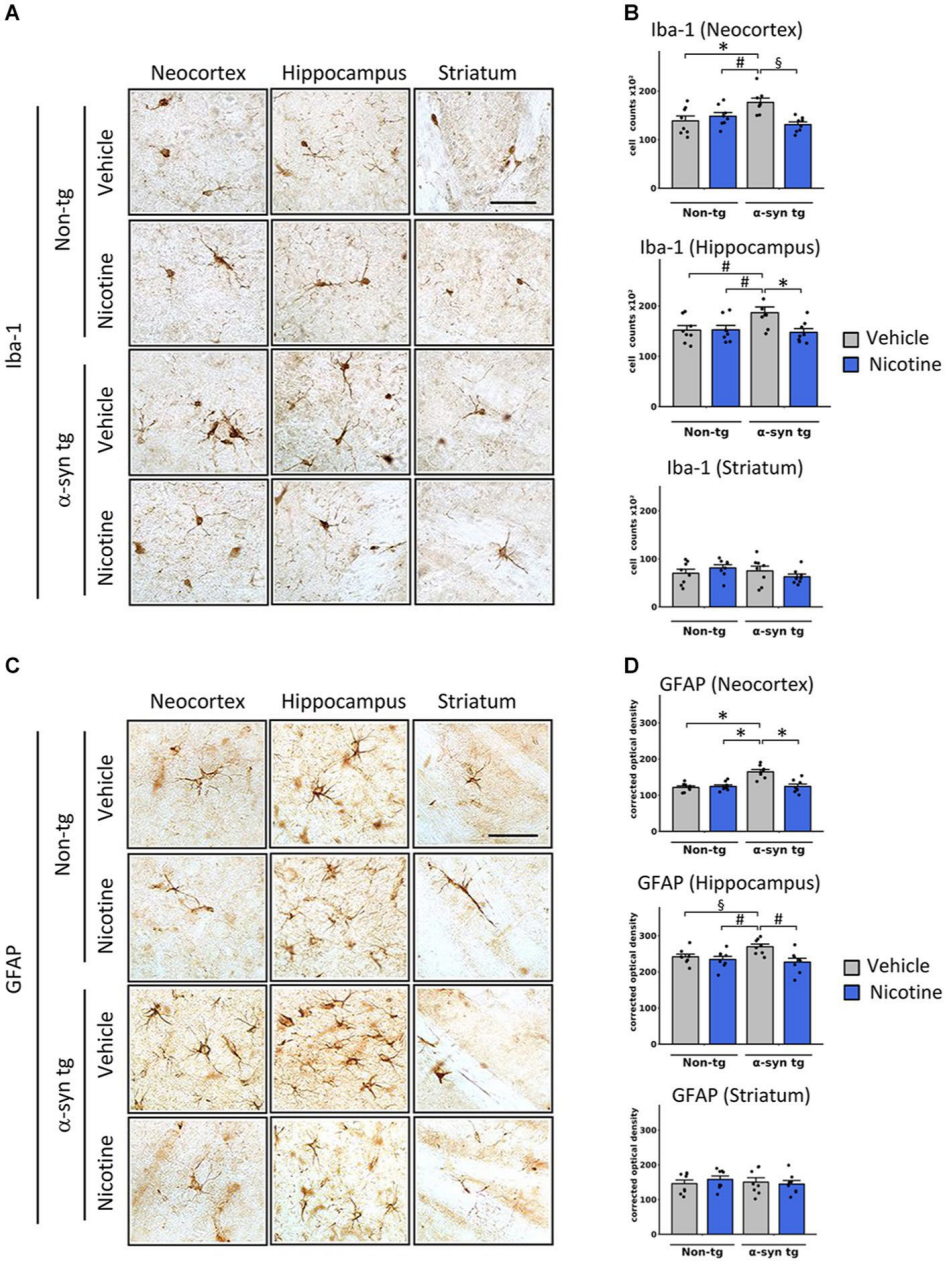
Effects of nicotine on microgliosis and astrogliosis in α-Syn tg mice. Twelve-month-old PDGFβ α-syn mice and non-tg mice were treated with nicotine (0.1 mg/kg intraperitoneal [IP]) or saline (= vehicle) twice daily for 2 weeks. Brain sections were immunohistochemically evaluated for neuroinflammation using the microgliosis marker anti-Iba-1 **(A,B)** or the astrogliosis marker anti-GFAP **(C,D)**. **(A)** Representative photomicrographs and **(B)** quantitation of Iba-1-positive cells in the neocortex, hippocampus, and striatum. The number of Iba-1-positive cells was significantly increased in the neocortex of saline-treated α-Syn tg mice relative to saline-treated non-tg mice, and nicotine treatment normalized the number of Iba-1-positive cells in α-Syn tg mice relative to saline-treated α-Syn tg mice. Nicotine treatment also reduced the number of Iba-1 cells in the hippocampus of α-Syn tg mice relative to saline-treated α-Syn tg mice. There were no significant changes in the striatum. ^§^*p* < 0.001. *p < 0.01 and ^♯^*p* < 0.05. **(C)** Representative photomicrographs and **(D)** quantitation of GFAP-positive cells in the neocortex, hippocampus, and striatum. GFAP immunoreactivity was significantly increased in the neocortex of saline-treated α-Syn tg mice relative to saline-treated non-tg mice, and nicotine treatment normalized the number of GFAP-immunoreactive cells in α-Syn tg mice relative to saline-treated α-Syn tg mice. Nicotine treatment also reduced GFAP immunoreactivity in the hippocampus of α-Syn tg mice relative to saline-treated α-Syn tg mice. There were no significant changes in the striatum. *p < 0.0001, ^♯^*p* < 0.01 and ^§^*p* < 0.05. In panels **(B,D)**, *N* = 8 mice/group, dots represent individual values and bars denote the mean ± standard error of the mean. Scale bar for all subpanels  = 50 μm.

We next assessed whether reduced α-Syn accumulation in tg mice was concomitant with attenuation of neuroinflammation in the neocortex, hippocampus, and striatum of tg mice by assessing levels of Iba-1 and glial fibrillary acidic protein (GFAP), which are validated immunomarkers if microglia and astroglia, respectively. In line with previous reports (Kim et al., 2018), microglia and astroglia were significantly increased in the neocortex of saline-treated α-Syn tg mice relative to saline-treated non-tg mice, indicating induction of a neuroinflammatory response in these mice due to α-Syn overexpression (Figure 2). Notably, nicotine treatment significantly reduced the expression levels of microglia (Figures 2A,B) and astroglia (Figures 2C,D) markers in the neocortex and hippocampus of α-Syn tg mice relative to saline treatment, while there were no significant changes in the striatum.

**Figure 3:**
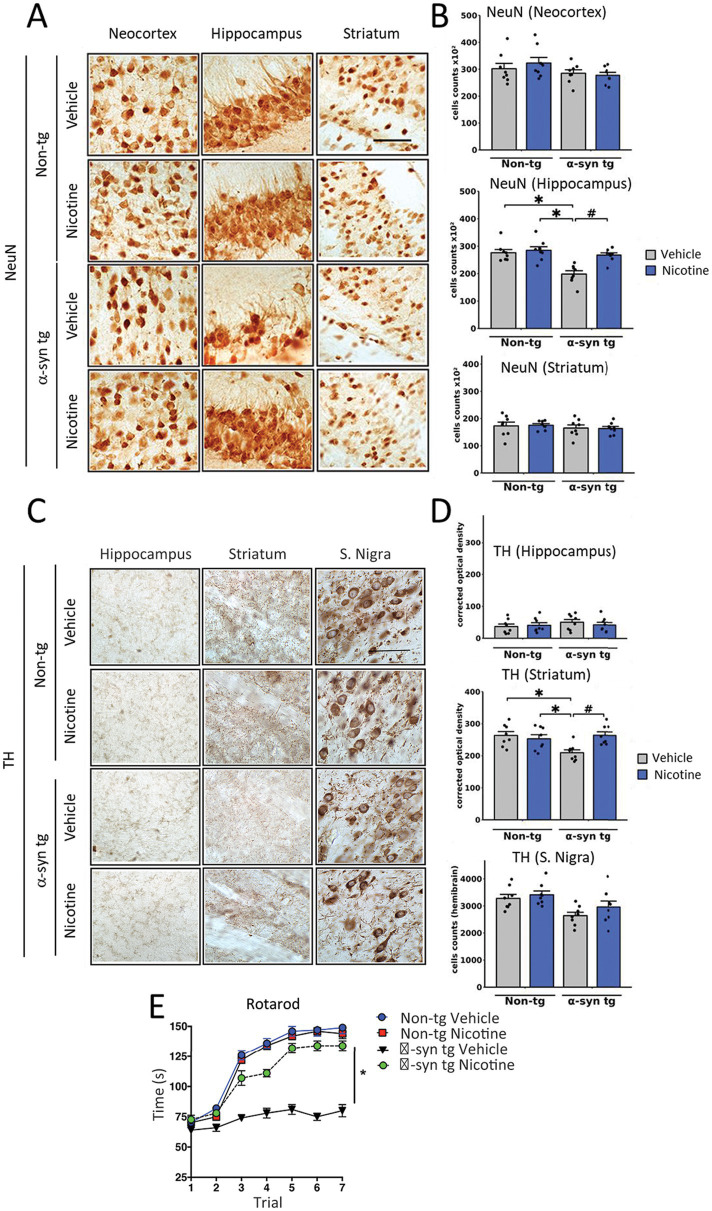
Effects of nicotine treatment on neurodegeneration and locomotor activity in α-Syn tg mice. Twelve-month-old PDGFβ α-Syn-tg and non-tg mice were treated with nicotine (0.1 mg/kg intraperitoneal [IP]) or saline (= vehicle) twice daily for 2  weeks. Brain sections were immunohistochemically evaluated for neurodegeneration with the neuronal marker NeuN **(A,B)** and the dopaminergic marker anti-TH **(C,D)**. **(A)** Representative photomicrographs and **(B)** quantitation of the number of neurons in the neocortex, hippocampus, and striatum. While the number of NeuN-positive cells was unchanged in the neocortex and striatum, saline-treated α-Syn tg mice had significantly fewer neurons in the hippocampus than saline-treated non-tg mice. Nicotine treatment normalized the number of NeuN-positive cells in the hippocampus of α-Syn tg mice relative to saline-treated α-Syn tg mice. *N* = 8/group. Scale bar = 50 μm. *p < 0.0001, ^♯^*p* < 0.001. **(C)** Representative photomicrographs and **(D)** quantitation of TH immunoreactivity in the substantia nigra (s. nigra), hippocampus, and striatum. While TH immunoreactivity was unchanged in the hippocampus and substantia nigra, saline-treated α-Syn tg mice exhibited significantly less immunoreactivity in the striatum than saline-treated non-tg mice. Nicotine treatment normalized TH immunoreactivity in the striatum of α-Syn tg mice relative to saline-treated α-Syn tg mice. *N* = 8/group. Scale bar = 50 μm. *p < 0.05, ^♯^*p* < 0.01. **(E)** The effect of nicotine treatment on locomotor activity was assessed using the rotarod test. Saline-treated α-Syn tg mice spent significantly less time on the rotarod than saline-treated non-tg mice. Nicotine-treated α-Syn tg mice remained on the rotarod for significantly longer than saline-treated α-Syn tg mice *p < 0.05, *N* = 8 mice/group. In panels **(B,D)**, dots represent individual values and bars denote the mean ± standard error of the mean.

To evaluate the effect of nicotine treatment on neurodegeneration, the number of NeuN-positive neurons was stereologically quantified across different brain regions (Figures 3A,B). The NeuN count was significantly decreased in the hippocampus of vehicle-treated α-Syn tg mice relative to vehicle-treated non-tg mice, with no significant neurodegeneration in the neocortex or striatum. Importantly, relative to saline treatment, treatment with nicotine significantly protected against neurodegeneration in the hippocampus of α-Syn tg mice.

We next investigated the effect of nicotine treatment on the dopaminergic system by stereologically quantifying the number of tyrosine hydroxylase (TH)-positive neurons in the substantia nigra and evaluating the levels of TH terminal projections in the striatum and hippocampus (Figures 3C,D). No significant degeneration of TH neurons was observed in the substantia nigra of saline-treated α-Syn tg mice relative to non-tg controls as previously published (Amschl et al., 2013). However, dopamine immunoreactivity was significantly reduced in the striatum of saline-treated α-Syn tg mice, and treatment with nicotine attenuated this selective neurodegeneration of TH-positive terminals. No significant difference in hippocampal TH immunoreactivity was observed between the saline- and nicotine-treated α-Syn tg mice.

To determine whether the observed changes in TH reactivity were accompanied by improvements in motor function, the rotarod test was applied to evaluate balance, grip strength, and motor coordination. In this standard and validated test of motor activity, the mouse is placed on a rod that rotates around an axis, and the mouse walks forward to remain upright and not fall off. The longer the latency time on the rod, the better the motor coordination of the mouse. After seven trials, saline-treated α-Syn tg mice remained on the rotarod for a significantly shorter period (around 75 s) than saline-treated non-tg mice (around 150 s), indicating motor deficits as we have previously reported (Amschl et al., 2013; Figure 3E). Strikingly, nicotine treatment for 2 weeks significantly enhanced the ability of α-Syn tg mice to remain on the rotarod for a longer time (around 130 s) than saline-treated α-Syn tg mice (around 75 s), indicating improved motor coordination. Taken together, these results demonstrate that nicotine treatment attenuates neuroinflammation and neurodegeneration in α-Syn tg mice and that the dopaminergic system may also be positively affected by nicotine administration.

### Differentiation of iPSCs into dopaminergic neurons

**Figure 4:**
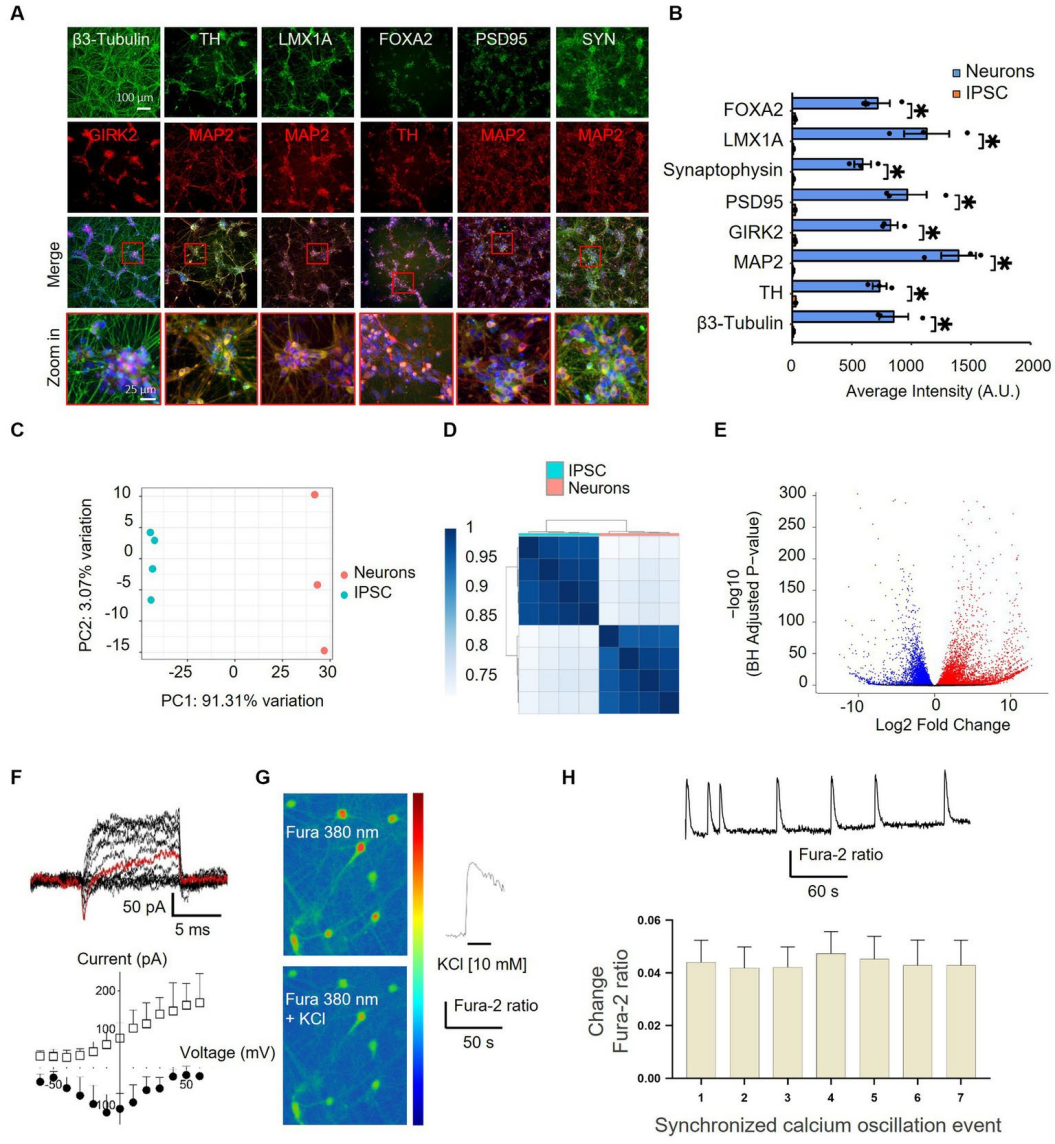
Characterization of differentiated iPSC-derived dopaminergic neurons by immunocytochemistry, RNA-sequencing, and electrophysiology. **(A)** Differentiated iPSCs were immunostained with neuronal (MAP2 and β3-Tubulin), synaptic (SYN and PSD95), and dopaminergic (TH, FOXA2, GIRK2, and LMX1A) markers after 35 DIV. The lowermost panel shows higher-magnification images of the red boxes overlaying the merged channel. FOXA2, forkhead box A2; GIRK2, G protein-activated inward rectifier potassium channel 2; LMX1A, LIM homeobox transcription factor 1, alpha; MAP2, microtubule-associated protein 2; PSD95, postsynaptic density protein 95; TH, tyrosine hydroxylase; SYN, synaptophysin. **(B)** Quantification of the immunostaining findings shown in panel **(A)** in both iPSCs and neurons indicates an upregulation of specific neuronal markers after differentiation. *N* = 3, dots represent individual values and bars denote the mean ± standard deviation. *p < 0.05. **(C,D)** RNAseq characterization of differentiated dopaminergic neurons. **(C)** Two principal component analyses (PC1 and PC2) of iPSCs (blue) and neurons (pink) show distinct expression patterns and within-group uniformity (*n* = 4). **(D)** Sample gene expression distance heatmap calculated using the Spearman correlation coefficients of all expressed genes in all four samples. **(E)** Volcano plot representing global differentially expressed genes (red and blue dots show significantly up- and downregulated genes, respectively, with adjusted *p* < 0.05) in iPSCs vs. neurons. **(F–H)** Assessment of the functionality of iPSC-derived neurons. **(F)** Current traces of responses to a voltage step protocol using a whole-cell automated patch-clamp setup at a holding potential of −80 mV confirmed the presence of Na^+^/K^+^ current in iPSC-derived neurons. The current response at −10 mV is highlighted in red. The corresponding current–voltage (I–V) relationship of the inward Na^+^ current (filled circles) and the outward K^+^ current (empty squares) is shown below (*n* = 4–5 cells). Vertical and horizontal lines represent the current and time scale of 50 pA and 1 ms, respectively. **(G)** iPSC-derived dopaminergic neurons displaying Fura-2 AM fluorescence, observed at 380 nm (unbound Fura), in a color-encoded map before (top right panel) and after (bottom right panel) 10 mM KCl-induced calcium intracellular increase (scale bar = 50 μm). A representative trace in response to 10 mM KCl stimulation is shown in the right panel. **(H)** Representative trace from one iPSC-derived neuron recorded by single-cell imaging shows spontaneous synchronized calcium oscillation events (top panel) and quantification (bottom panel) of events recorded from different iPSC-derived neurons in the same well (*n* = 17), bars denote mean ± standard deviation.

To gain insights into the effect of nicotine on dopaminergic neurons and the implication of the nicotinic signaling pathway in mediating neuroprotective effects, we employed a humanized *in vitro* model of synucleinopathy. iPSCs were first differentiated into mature dopaminergic neurons by following a commercially available “floor plate-based” protocol (Ono Y. et al., 2007; Fasano et al., 2010; Kriks et al., 2011; Li et al., 2011; Xi et al., 2012; Supplementary Figure 2A). Immunostaining confirmed the expression of the pluripotency markers in iPSCs prior to differentiation (Bharathan et al., 2017; Supplementary Figure 2B), and DNA sequencing showed no mutations of known severe impact on gene function in the A18945 iPSC line used in this study. Mid-brain-specified Floor Plate progenitor (FPp cells) were efficiently generated after 10 days of iPSC specification, as evidenced by increased expression of FPp markers in most cells (Supplementary Figure 2C). These were then expanded and directed into neurons after passing through a stage of spheroid body formation. At 35 days *in vitro* (DIV), the expression of key neuronal, synaptic, and dopaminergic (Inanobe et al., 1999; Kittappa et al., 2007) lineage markers was confirmed by immunostaining (Figures 4A,B). At this stage, TH-positive cells represented 64.5 ± 2.7% of the cells per well, thereby confirming substantial enrichment of dopaminergic neurons in these cultures.

To further validate and characterize the induction of a neuronal phenotype, next-generation transcriptome sequencing (RNA-seq) analysis was performed on iPSCs before and after applying the differentiation protocol. The PCA results indicated a reset in gene expression at 35 DIV and demonstrated that iPSCs and iPSC-derived neurons clustered apart and showed within-group uniformity (Figures 4C,D). A total of 9,910 differentially expressed mRNAs were identified, with 5,649 upregulated and 4,261 down-regulated transcripts (Figure 4E). Among the post-differentiation upregulated genes were many immature and mature neuronal and dopaminergic markers and neurotransmitter receptors, and—as expected—the differentiation protocol “turned off” the expression of known stem cell markers in differentiated neurons (Supplementary Figure 3). Similarly, GO enrichment analysis highlighted a significant upregulation of genes associated with synaptic function and neuronal projection post-differentiation (Supplementary Figure 2D). Notably, clusters of genes implicated in glutamatergic and GABAergic synapse formation were also significantly induced, indicating the additional presence of neurons exhibiting glutamatergic and GABAergic phenotypes within these cultures.

To assess the functional maturation of iPSC-derived neurons, electrophysiological recordings and assessment of intracellular calcium changes were performed. At 38 DIV, the differentiated neurons exhibited transient inward sodium currents and sustained outward potassium currents, as measured with an automated patch clamp (Figure 4F). Moreover, image-based assessment of intracellular calcium changes provided evidence of membrane depolarization and increased intracellular calcium levels following treatment with potassium chloride (Figure 4G) as well as synchronized spontaneous calcium oscillations (Figure 4H). Together, these results indicate the differentiation of iPSCs into functional dopaminergic, GABAergic, and glutamatergic neurons starting at 35 DIV, with substantial enrichment of dopaminergic neurons within these cultures.

### Establishment of a humanized *in vitro* dopaminergic neuronal model of synucleinopathy

To generate a humanized neuronal model of α-Syn-provoked neurodegeneration, we first sought to overexpress human α-Syn in mature iPSC-derived dopaminergic neurons using lentiviruses. As a primary readout for assessing the effect of α-Syn on the viability and neuritic outgrowth of iPSC-derived neurons, we deployed xCELLigence™ Real-Time Cell Analyzer (RTCA), as this readout allows non-invasive, label-free, and highly sensitive assessment of cellular adhesion in real-time and in a medium-throughput manner by measuring changes in electron flow between gold electrodes fused to the bottom of the xCELLigence E-plates. The suitability of this system to measure neuronal toxicity was validated by assessing the effect of carbonyl cyanide m-chlorophenyl hydrazone (CCCP), a compound that is known to induce neuronal toxicity by inhibiting oxidative phosphorylation (Summers et al., 2014). CCCP treatment provoked a significant drop in neuronal adhesion (Supplementary Figures 4A,B), which was concomitant with a decrease in cellular ATP levels (Supplementary Figure 4C) and an increase in nuclear TUNEL staining, an indicator of apoptosis (Supplementary Figure 4D), thereby validating the utility of the RTCA system to sensitively assess toxic effects in neurons.

**Figure 5:**
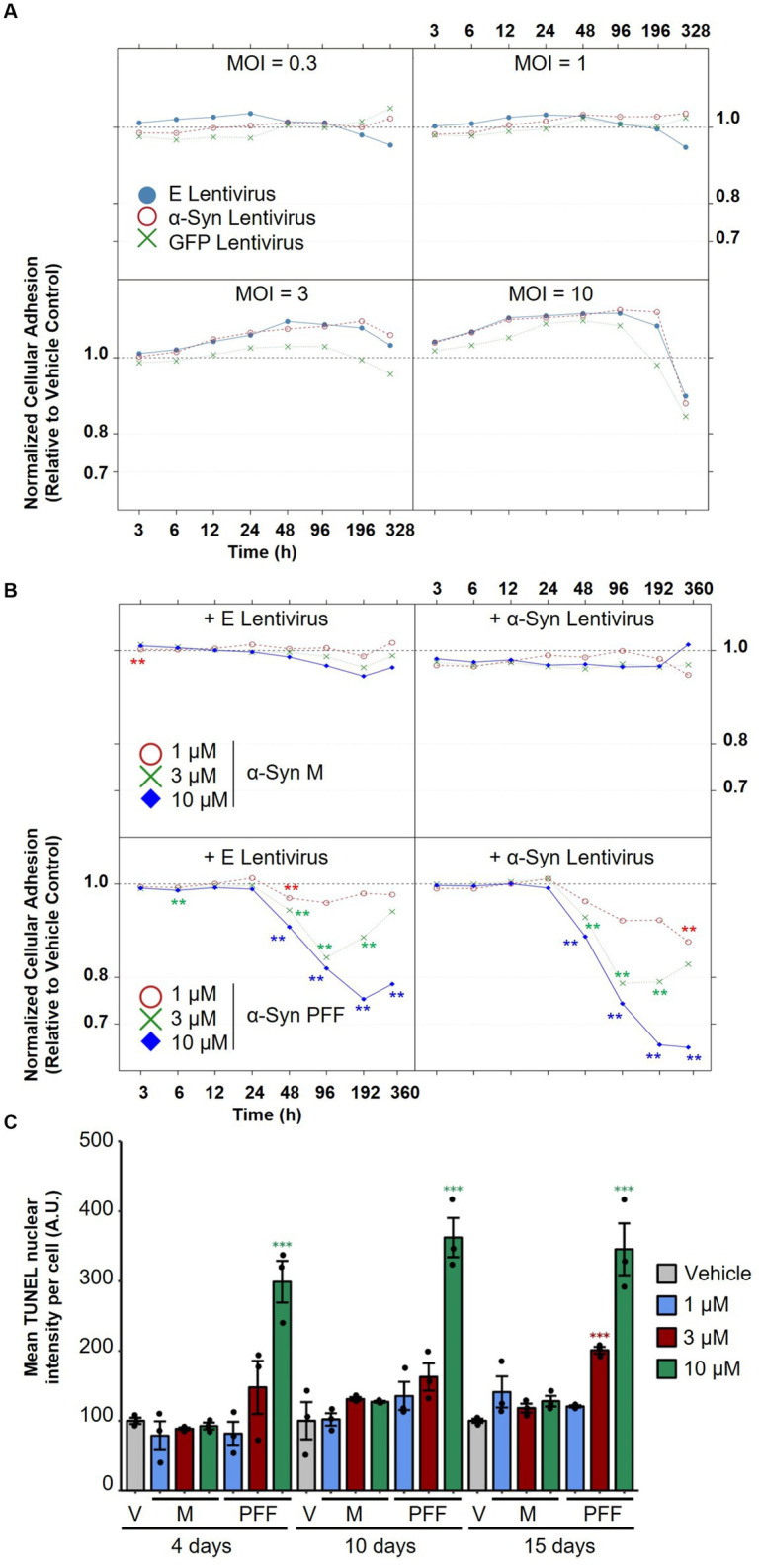
Assessment of the effect of α-Syn lentiviral overexpression versus treatment with α-Syn PFF on dopaminergic neuron degeneration. **(A)** iPSC-derived neurons were treated with increasing titers (MOI 0.3, 1, 3, and 10) of lentiviruses encoding human α-Syn, empty vectors or GFP as controls, and normalized cellular impedance relative to the vehicle control was monitored over time using the xCELLigence® Real-Time Cell Analyzer (RTCA) system. **(B)** iPSC-derived neurons were first treated with lentiviruses encoding human α-Syn or empty vectors as controls at MOI 5, and then treated after 2 days with increasing concentrations (1, 3, 10 μM) of α-Syn PFFs or α-Syn M as controls, and normalized cellular impedance relative to the vehicle control was monitored over time using the RTCA system. **(C)** Assessment of the effect of treatment with different concentrations of α-Syn PFF or α-Syn M over time on mean TUNEL nuclear intensity per cell using automated fluorescent quantification. In panels **(A–C)**, *N* = 3 independent experiments. In panel **(C)**, dots represent individual values and bar plots represent mean ± standard deviation. A.U.: arbitrary unit; V: vehicle control; M: α-Synuclein monomers; PFF: α-Synuclein pre-formed fibrils; E: empty vector; MOI: multiplicity of infection. **p-values <0.01, ***p-values <0.001.

To evaluate the effect of α-Syn expression on neuronal viability using the RTCA system, mature neurons (35 DIV) were treated with increasing titers of lentiviruses encoding human α-Syn or with viruses encoding empty vectors as controls. To further control for non-α-Syn-specific toxicity due to increased overexpression, separate cultures were treated with lentiviruses encoding GFP. As shown in Figure 5A, none of the lentiviral preparations caused any toxicity, measured by the RTCA system upon treatment at 0.3, 1, and 3 MOI, for up to 2 weeks of overexpression in iPSC-derived dopaminergic neurons. However, treatment with the highest dose of the viruses (MOI 10) led to toxicity, which was judged to be non-specific, as it was observed upon treatment with viruses encoding human α-Syn and viruses encoding empty vector controls or GFP. This result was not too surprising, as many of the cellular models relying on α-Syn overexpression have shown limited success in provoking toxicity in cell lines and primary neurons, as we recently reviewed (Fares et al., 2021).

We next evaluated whether treatment of differentiated IPSC-derived neurons with increasing doses of α-Syn PFF would cause more pronounced pathological changes, as this strategy has been successful in inducing endogenous α-Syn aggregation, toxicity, and mitochondrial dysfunction in cell lines and neonatal murine neurons (Volpicelli-Daley et al., 2011; Wang et al., 2019; Mahul-Mellier et al., 2020; Fares et al., 2021). Synthetic α-Syn PFF were generated as previously described (Polinski et al., 2018), and their integrity and amyloidogenic content were verified with a battery of techniques including SDS-PAGE, mass spectrometry, assessment of solubility, ThT binding, and transmission electron microscopy (TEM) (Supplementary Figure 5). To assess whether treatment with α-Syn PFF—alone or in conjunction with lentiviral overexpression of α-Syn—would provoke dopaminergic toxicity, mature neurons (35 DIV) were first treated with lentiviruses encoding human α-Syn (or empty vectors as controls) and then treated after 2 days with increasing concentrations of α-Syn PFF (or α-Syn M as controls). The medium was completely changed after 2 days of treatment, and half changed every 2 subsequent days to allow prolonged incubation for up to 15 days of treatment. Importantly, we observed that treatment with α-Syn PFF provoked a significant decrease in impedance in a time- and dose-dependent manner, indicating a toxic effect (Figure 5B). Moreover, lentiviral overexpression of α-Syn did not affect the toxicity induced by α-Syn PFF. The drop in impedance observed upon α-Syn PFF treatment with or without lentiviruses was similar and evident starting at 2 days post-treatment and reached its lowest levels at 4 days post-treatment with 1 μM and 3 μM α-Syn PFF and at 8 days of treatment with 10 μM α-Syn PFF. Notably, treatment with 1 μM PFF led to an overall minor drop in neuronal adhesion, and partial recovery was observed in the 3 μM α-Syn PFF condition after 15 days of treatment, possibly reflecting a reversible neurite shortening effect in this condition. On the other hand, treatment with the highest dose of α-Syn PFF induced the most severe drop in impedance compared with the other tested doses, with no clear recovery observed. Indeed, quantification of neuronal apoptosis by the TUNEL assay showed no cell death at 1 μM α-Syn PFF treatment at any time point, while, upon treatment with 3 and 10 μM α-Syn PFF, apoptosis was observed at 15 days (3 μM) and at 4, 10, and 15 days (10 μM) of treatment, respectively (Figure 5C). In contrast, similar concentrations of α-Syn M, used as controls, did not induce any cell death in the treated neuronal cultures.

### Nicotine and anatabine exhibit neuroprotective effects in a humanized neuronal model of synucleinopathy

**Figure 6:**
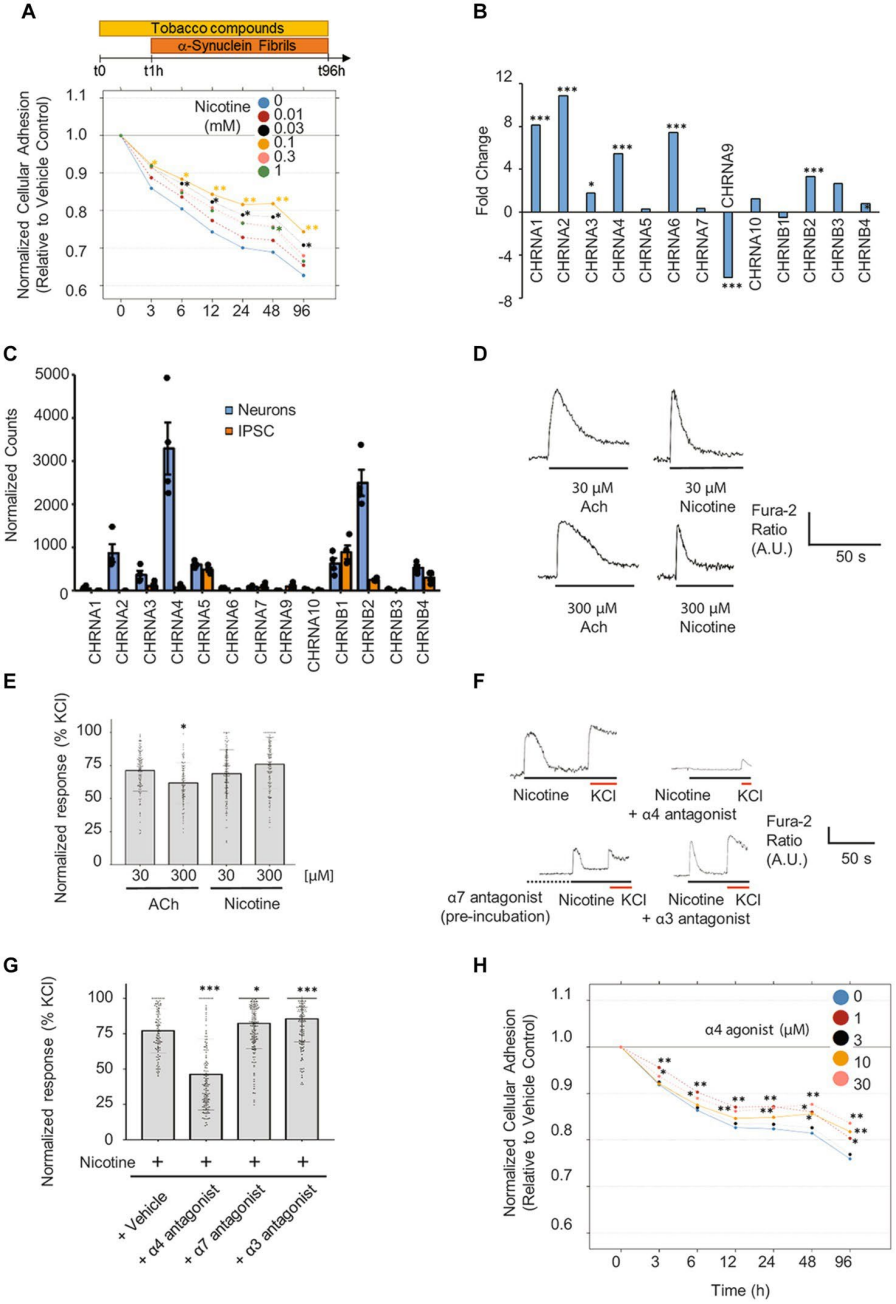
Pretreatment with nicotine or α4 agonist attenuates α-Syn PFF-provoked degeneration of differentiated iPSC-derived neurons. **(A)** Mature iPSC-derived neurons were pretreated for 1 h with increasing doses of nicotine (from 0.01 to 1 mM) followed by a 4-day co-exposure to α-Syn PFF (3 μM). Assessment of neuronal adhesion throughout the experiment using the xCELLigence™ Real-Time Cell Analyzer system shows that nicotine pre-treatment attenuated α-Syn PFF-provoked toxicity mostly at 0.03 and 0.1 mM. *N* = 3 independent experiments. **(B)** Fold-change in expression levels and **(C)** normalized counts of transcripts detected in neurons and iPSCs are shown. Count normalization was done using DESeq2’s median of ratios method. *n* = 4 biological replicates. **(D)** Representative traces in response to nicotine or acetylcholine (ACh) treatment at two different concentrations. **(E)** Scatter plot illustrating the Fura-2 ratio peak calcium changes, quantifying responses normalized to KCl (10 mM) (*n* = 97–233 cells). **(F)** Representative Fura-2 traces from dopaminergic neurons in response to nicotine (100 μM) treatment with or without different specific nAChR antagonists. The α4 antagonist (DMAB, 100 μM) and α3 antagonist (SR16584, 100 μM) were coapplied with nicotine (100 μM). The α7 antagonist (methyllycaconitine (MLA), 40 nM) was first preincubated (2 min, dotted line) before addition of 100 μM nicotine. **(G)** Scatter plot illustrating the Fura-2 ratio peak calcium changes, quantifying responses normalized to KCl (10 mM) (*n* = 102–194 cells). **(H)** Mature neurons were pretreated for 1 h with increasing doses of the α4-specific agonist AZD1446 (from 1 to 30 μM), followed by a 4-day exposure to α-Syn PFF (3 μM). Assessment of neuronal adhesion throughout the experiment showed that AZD1446 attenuates α-Syn PFF toxicity mostly at 1 and 30 μM. *N* = 3 independent experiments. *p < 0.05, **p < 0.01, ***p < 0.001 in all subpanels.

After establishing a humanized neuronal model of synucleinopathy, we assessed the effects of nicotine on α-Syn PFF-induced neurodegeneration. To this end, mature neurons were pretreated for 1 h with increasing concentrations of nicotine and then co-treated for 4 days with α-Syn PFF. In this assay, we chose to treat neurons with the intermediate concentration of α-Syn PFF (3 μM) to provoke an intermediate degree of degeneration that could potentially be rescued. We selected a post-exposure duration of 4 days based on our observation that maximum impedance decreases at this time point, which is concomitant with the appearance of dystrophic and ballooned neurites (Supplementary Figure 6A), a phenotype that is well established for α-Syn-provoked neurotoxicity in neuronal and *in vivo* models (Oueslati et al., 2012; Koch et al., 2015). Notably, pretreatment with nicotine significantly attenuated neuronal degeneration as measured by RTCA (Figure 6A), with neuroprotective effects observed at low to intermediate concentrations (0.03–0.1 mM). Importantly, this effect was concomitant with significant rescue of the drop in cellular ATP levels (Supplementary Figure 6B), as well as with attenuated neuritic pathology which was observed following treatment with α-Syn PFF (Supplementary Figure 6A), thereby indicating that nicotine pretreatment attenuates the toxic effects of α-Syn PFF in this model.

To control for possible effects of nicotine pretreatment on neuronal adhesion or ATP levels independently of suppressing α-Syn PFF-mediated phenotypes, we evaluated mature iPSC-derived neurons exposed to different doses of nicotine without any α-Syn PFF co-treatment. As shown in Supplementary Figures 6B,C, nicotine treatment alone had no significant enhancing effect on ATP levels or cellular impedance measurements by RTCA. In contrast, at later time points nicotine-treated mature iPSC-neurons showed a tendency for decreased overall adhesion, thereby ruling out the possibility that nicotine could be ameliorating neuronal adhesion independently of α-Syn PFF-mediated phenotypes.

As nicotine altered the neurotoxic potential of aggregated α-Syn in tg mice and iPSC dopaminergic neurons, we sought to investigate the mechanism via which nicotine might reduce neurodegeneration. We first examined whether nicotine pretreatment might directly interfere with α-Syn PFF uptake into iPSC neurons. Importantly, high-resolution confocal imaging revealed neuronal internalization of α-Syn PFF within many of the neurons at 4 days of treatment, independent of concentration (Supplementary Figure 7). Moreover, quantification of neurons with internalized α-Syn PFF did not reveal any significant differences among the groups, indicating that nicotine does not attenuate the uptake of α-Syn PFF into dopaminergic neurons (Supplementary Figure 8A).

As nicotine is a known ligand to nAChR (Alijevic et al., 2020), and it has been previously suggested that nAChR may mediate protective effects in toxin models of neurodegeneration (Perez et al., 2010; Huang et al., 2011; Perez and Quik, 2011; Quik and Wonnacott, 2011; Quik et al., 2013), we investigated whether activation of nAChR may mediate neuroprotective effects in our model. First, we evaluated RNA expression levels of the complete panel of nicotinic receptors in iPSC neurons by RNA sequencing. We detected significant upregulation of several nAChR subunits (CHRNA1-4, CHRNA6, CHRNB2, CHRNB4) in dopaminergic neurons relative to iPSCs, with subunits that form α4β2 receptors being the most abundantly expressed (Figures 6B,C). We then evaluated whether the expressed subunits assemble into functional nAChR in iPSC-derived neurons. To this end, we screened for the ability of specific agonists to activate nicotinic receptors containing the α3, α4, α6, and α7 subunits using calcium imaging (Supplementary Table 3). The functionality of nicotinic receptors containing other subunits was not assessed because of either the lack of specific pharmacological modulators or their very low abundance in iPSC-derived neurons according to our RNAseq data. As shown in Supplementary Figures 8B,C, treatment with each of the tested nicotinic receptor agonists at 10 μM triggered a neuronal response, indicating the presence of functional nicotinic receptors comprising the α3, α4, α6, and α7 subunits. Interestingly, only the responses to α4 and α7 agonists were dose-dependent in these cultures (Supplementary Figure 8C).

Next, we evaluated the ability of nicotine to elicit calcium responses in these neurons. Notably, nicotine treatment provoked a spike in calcium levels that was comparable to the response elicited by acetylcholine (Supplementary Figure 8D; Figures 6D,E). To determine the main nAChR subunits activated by nicotine in iPSC-derived neurons, we cotreated cultures with nicotine and specific antagonists against the α3, α4, and α7 subunits. Importantly, only the α4 antagonist reduced the nicotine-mediated calcium response, indicating that nAChR containing α4 subunits are probably the main receptors activated by nicotine in our iPSC-derived neuronal cultures (Figures 6F,G). Based on this finding, we screened the ability of a panel of 10 tobacco alkaloids and nicotinic agonists to modify α-Syn-provoked neurodegeneration in iPSC neurons. Cultures of mature dopaminergic neurons (35 DIV) were pretreated for 1 h with increasing doses of different compounds (Supplementary Table 2) and then exposed to 3 μM α-Syn PFF for 4 days. Strikingly, only the α4β2 nAChR-specific agonist AZD1446 and the tobacco alkaloid anatabine—which is also a potent partial agonist of α4β2 nAChR (Villar-Pique et al., 2016; Alijevic et al., 2020)—significantly attenuated neuronal degeneration at low and high concentrations, as evidenced by real-time assessment of neuronal adhesion (Figure 6H; Supplementary Figure 9). Collectively, these results indicate that activation of α4β2 receptors mediates nicotine’s neuroprotective effects against α-Syn PFF-provoked degeneration of iPSC-differentiated neurons.

## Discussion

In this study, we investigated whether nicotine attenuates neuropathology in animal and neuronal models of synucleinopathy. Our findings demonstrate a neuroprotective effect of tobacco alkaloids against α-Syn aggregation and toxicity *in vivo* as well as in human dopaminergic neurons treated with extracellular α-Syn PFFs. Moreover, our results implicate α4β2 nicotinic receptors in mediating the neuroprotective effects of nicotine against α-Syn pathology.

In tg mice overexpressing α-Syn under the PDGFα promoter, nicotine treatment attenuates several pathological features including α-Syn aggregation, accumulation of phosphorylated α-Syn species, neuroinflammatory responses, neurodegeneration in different brain regions, and motor coordination deficits. Our results describing the neuroprotective effects of nicotine *in vivo* are in line with those of a multitude of previous studies proposing a beneficial effect of this alkaloid in different animal models of PD. In these investigations, the neurotoxin 6-OHDA or 1-methyl-4-phenyl 1,2,3,6-tetrahydropyridine (MPTP) was typically used to induce degeneration of dopaminergic neurons and mimic PD in mice, rats, and non-human primates. Notably, in most of these studies, nicotine administration reduced dopaminergic neuron degeneration, protected against locomotor activity deficits, and reduced L-DOPA-provoked dyskinesia (Costa et al., 2001; Ryan et al., 2001; Meshul et al., 2002; Parain et al., 2003; Quik et al., 2006; Visanji et al., 2006; Park et al., 2007; Zhang et al., 2014). Our findings are also in agreement with previous publications showing that nicotine attenuates neuroinflammation, including lipopolysaccharide-induced microglial activation (Noda and Kobayashi, 2017) and inflammatory brain responses in mouse models of experimental autoimmune encephalomyelitis (Nizri et al., 2009).

Our *in vivo* results are partially in line with those of a previous study by Subramaniam and colleagues that investigated the effects of nicotine treatment in tg mice overexpressing human α-Syn under the Thy-1 promoter and reported improvement of cognitive impairment after chronic nicotine treatment (Subramaniam et al., 2018). However, the authors did not observe any effect of nicotine on motor deficits, α-Syn aggregation, neuroinflammation, or neurodegeneration. These discrepancies could be inherent to differences in the tg mouse line used, as Thy-1 mice exhibit a more pronounced phenotype relative to the D-line used in our study, with higher levels and different patterns of α-Syn accumulation. Whereas D-line mice show a mild phenotype, with α-Syn accumulation in the neocortex, limbic system (amygdala, hippocampus, thalamus, and hypothalamus), and olfactory regions, Thy-1 mice exhibit α-Syn accumulation in synapses and neurons throughout the brain, including the thalamus, basal ganglia, substantia nigra, and brainstem. The differences between the two studies might also be linked with variability in the mode and duration of nicotine treatment. In the Thy-1 tg experiments, nicotine was continuously administered via a subcutaneous osmotic pump at doses far higher than those used in our experiments (up to 20 times higher), and analysis was performed after longer time points (up to 6 months versus 2 weeks). Moreover, neuroinflammation assessments were performed in different brain regions in the two studies; we examined Iba1 staining in the striatum, neocortex, and hippocampus, while Subramaniam et al. assessed neuroinflammation in the substantia nigra. Finally, the age and sex of the mice in the two studies were different.

After assessing the effects of nicotine *in vivo*, we observed that nicotine pretreatment attenuates α-Syn PFF-induced toxicity in a humanized iPSC-derived dopaminergic model and that activation of α4β2 nicotinic receptors might be involved in mediating neuroprotective effects. Although few studies have assessed the effect of nicotine administration in cell culture models of PD, many of these reported beneficial effects. For example, nicotine was shown to protect against toxin-induced toxicity in mesencephalic dopaminergic neurons (Quik and Jeyarasasingam, 2000) and SH-SY5Y cells (Riveles et al., 2008), to attenuate the unfolded protein response in mouse ventral midbrain neurons exhibiting tunicamycin-evoked endoplasmic reticulum stress (Srinivasan et al., 2016), and to protect against α-Syn overexpression-induced toxicity in differentiated LUHMES cells (Kardani et al., 2017). However, no previous studies evaluated the effect of nicotine on extracellular α-Syn PFF-provoked neurotoxicity, as we have in this work.

Our results also show that other tobacco alkaloids, such as anatabine, could be also involved in conferring neuroprotective effects. Indeed, pretreatment with anatabine significantly attenuated α-Syn PFF-provoked toxicity. This effect for anatabine has not been reported to date; only a few studies have described the anti-inflammatory effects of anatabine and its sister alkaloid anabasine in animal models of Alzheimer’s disease, with the effects mostly mediated by suppressing phosphorylation of STAT3 and p65 (also known as RELA) (Paris et al., 2013; Verma et al., 2015). Notably, although anatabine treatment offered similar levels of neuroprotection as nicotine treatment in our model, it elicited a significantly lower calcium response (data not shown), possibly suggesting that these compounds are neuroprotective via different mechanisms. Further studies are needed to unravel these mechanisms and dissect the relative roles of different alkaloids in mediating potential neuroprotective effects.

Alkaloids were reported to slow degeneration by upregulating the expression of anti-apoptotic proteins (Dasgupta et al., 2006) and P450 cytochromes that can detoxify neurotoxins (Miksys and Tyndale, 2006), but other studies suggested a potential direct effect of nicotine on α-Syn aggregation, with inhibition of recombinant α-Syn protein aggregation at intermediate to high nicotine concentrations (0.5–1 mM) (Ono K. et al., 2007; Hong et al., 2009), as well as of ectopically expressed α-Syn in yeast (Kardani et al., 2017). Moreover, because nicotine administration was found to directly stimulate dopamine release *in vivo* (Quik et al., 2008), a merely symptomatic effect is also plausible in animal models. In this study, nicotine treatment did not directly interfere with the uptake of exogenously added α-Syn PFF by dopaminergic neurons, but it did activate α4β2 nicotinic receptors. This event could trigger a subsequent cellular response that promotes neuritic outgrowth and survival. Our results implicating α4β2 nicotinic receptors in mediating nicotine’s beneficial effects in the context of PD are in accordance with previous studies involving clinical samples and preclinical models. Receptor studies using radiolabeled nicotine, methylcarbachol, and epibatidine revealed that PD patients exhibit a 30–75% decline in nicotinic receptors in the caudate putamen and substantia nigra, correlating with the observed nigrostriatal damage. The level of expression of both α6β2 and α4β2 nAChR were reportedly decreased in the brains of PD patients (Quik et al., 2008). Furthermore, cyclic voltammetry experiments in nicotine-treated Parkinsonian rats demonstrated that α4β2 nicotinic receptors mediate neuroprotection against nigrostriatal damage and reduction in L-DOPA-induced dyskinesias (Perez et al., 2010; Huang et al., 2011; Perez and Quik, 2011). Acute nicotine treatment of α4 nAChR subunit-knockout mice failed to inhibit methamphetamine- and 6-OHDA-induced neurodegeneration, highlighting the critical role of this subunit in mediating the beneficial effects of nicotine (Ryan et al., 2001; Quik et al., 2013). Likewise, nicotine was postulated to exert its neuroprotective effects in cellular models by chaperoning α4β2 receptors to the cell surface and suppressing endoplasmic reticulum stress and the unfolded protein response, which together can enhance cell survival (Srinivasan et al., 2011, 2012). Importantly, a recent study utilizing mouse primary neurons and iPSC-derived dopaminergic neurons also showed that nicotine protects against neuronal injury provoked by glucose deprivation and reduces α-Syn accumulation, possibly through activation of the dopamine D3 receptor, in addition to the involvement of the β2 subunit of nAChR (Bono et al., 2019).

## Conclusion

Taken together, our results show that nicotine exerts protective effects against α-Syn-provoked pathology *in vivo* and in human iPSC-derived neurons. This study establishes the utility of iPSC-derived neurons in modeling α-Syn-provoked pathology and assessing the potential beneficial effects of candidate compounds. In parallel to its known direct inhibitory effect on α-Syn aggregation, which may explain some of the observed effects of nicotine in the *in vivo* model, our observations also implicate activation of α4β2 nicotinic receptors in mediating nicotine’s effect. Further studies are needed to elucidate the protective signaling pathways triggered by α4β2 nAChR, as this could hold promise towards developing novel molecules with disease-modifying potential for synucleinopathies based on the structure and properties of nicotinic alkaloids.

## Data availability statement

The datasets presented in this study can be found in online repositories. The names of the repository/repositories and accession number(s) can be found at: https://www.ncbi.nlm.nih.gov/ bioproject/PRJNA613205.

## Ethics statement

Ethical approval was not required for studies on humans in accordance with the local legislation and institutional requirements because only commercially available established cell lines were used. The animal study was approved by Institutional Animal Care and Use Committee of the University of California San Diego. The study was conducted in accordance with the local legislation and institutional requirements.

## Author contributions

MF was involved in conceptualization, methodology, formal analysis, investigation, resources, visualization, project administration, and writing the original draft, reviewing and editing. OA contributed to methodology, formal analysis, investigation, resources, visualization, and writing – reviewing and editing. SJ contributed to investigation, resources, and data curation. CO contributed to methodology, formal analysis, investigation, visualization, and writing the original draft. MH performed methodology, formal analysis, investigation, visualization, and writing an original draft as well as visualization. AK performed a formal analysis, visualization, and writing – reviewing and editing. AA contributed to investigation and resources. RD, DP, and CN were involved in investigation. JB performed investigation and formal analysis. EG curated data and supervised. NS was involved in visualization, formal analysis, supervision, and writing – reviewing and editing. DM contributed to methodology, supervised, writing – reviewing and editing. ER contributed to methodology, investigation, project administration, and supervised. CK contributed to methodology, investigation, and formal analysis. RR was involved in conceptualization, methodology, writing – reviewing and editing, and funding acquisition. JH was involved in funding acquisition and supervision. MP performed funding acquisition. EM was involved in in conceptualization, methodology, formal analysis, visualization, supervision, writing, reviewing, and editing, and funding acquisition. CM was involved in in conceptualization, methodology, visualization, supervision, writing – reviewing and editing, and project administration. All authors contributed to the article and approved the submitted version.

## Funding

Philip Morris International funded only the *in vitro* experiments described in this study. The *in vivo* studies were performed at UCSD with an internal funding source.

## Conflict of interest

OA, SJ, AK, RD, DP, CN, JB, EG, NS, DM, and CM are employees of Philip Morris International. MP was a PMI employee at the time of the study and is now retired. JH was a PMI employee at the time of the study and is now an employee of Vectura Fertin Pharma. MF was a PMI employee at the time of the study and is now a co-founder and director of R&D at ND Biosciences, a company developing next-generation therapeutics and diagnostics for neurodegenerative diseases.

The remaining authors declare that the research was conducted in the absence of any commercial or financial relationships that could be construed as a potential conflict of interest.

## Correction note

A correction has been made to this article. Details can be found at: 10.3389/fnins.2025.1662162.

## Publisher’s note

All claims expressed in this article are solely those of the authors and do not necessarily represent those of their affiliated organizations, or those of the publisher, the editors and the reviewers. Any product that may be evaluated in this article, or claim that may be made by its manufacturer, is not guaranteed or endorsed by the publisher.
