# Correction: Nicotine-mediated effects in neuronal and mouse models of synucleinopathy

**DOI:** 10.3389/fnins.2025.1662162

**Published:** 2025-08-01

**Authors:** Mohamed Bilal Fares, Omar Alijevic, Stephanie Johne, Cassia Overk, Makoto Hashimoto, Athanasios Kondylis, Anthony Adame, Remi Dulize, Dariusz Peric, Catherine Nury, James Battey, Emmanuel Guedj, Nicolas Sierro, Damian Mc Hugh, Edward Rockenstein, Changyoun Kim, Robert A. Rissman, Julia Hoeng, Manuel C. Peitsch, Eliezer Masliah, Carole Mathis

**Affiliations:** ^1^PMI R&D, Philip Morris Products S.A., Neuchâtel, Switzerland; ^2^Department of Neurosciences, University of California, San Diego, San Diego, CA, United States

**Keywords:** synucleinopathy, induced pluripotent stem cell (iPSC), transgenic mice, nicotine, nicotinic acetylcholine receptors (nAChR), neuroprotection

In the published article, there was an error in Figure 3C as published whereby micro-repeats appeared in the panel corresponding to the hippocampus of the vehicle non-tg control image. The corrected Figure 3 and its caption appear below.

**Figure 3:**
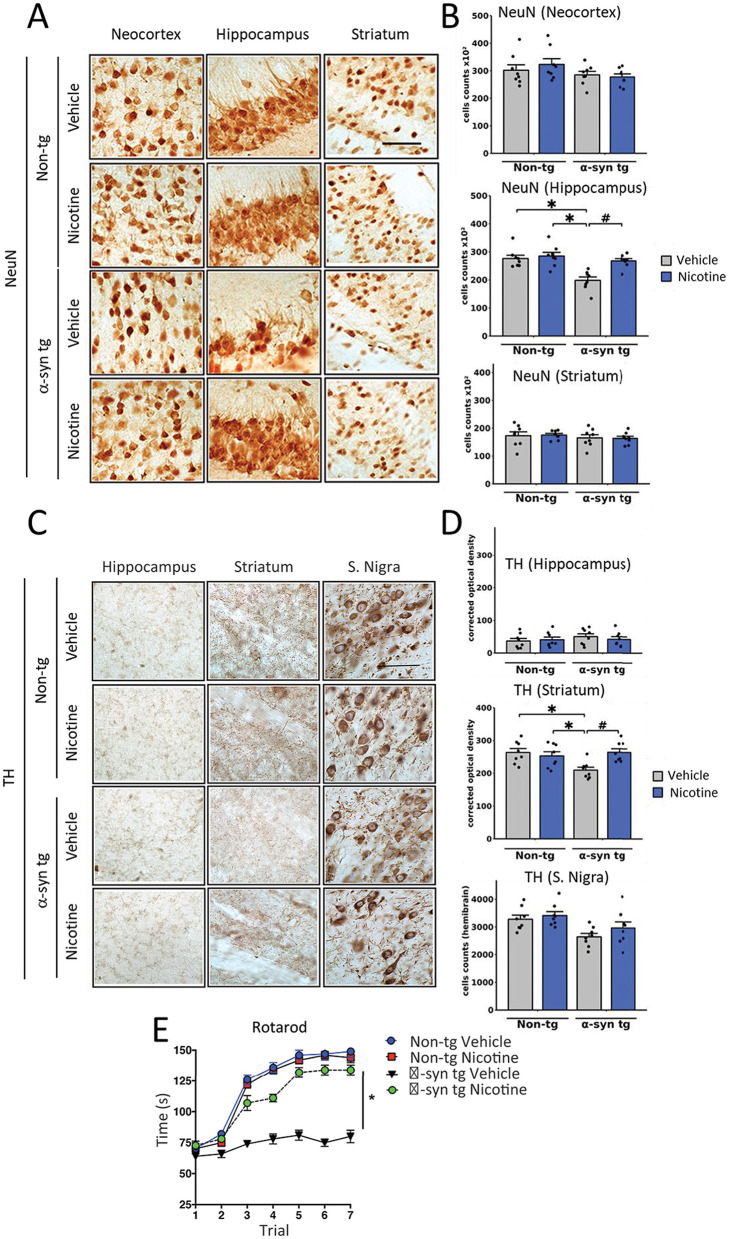
Effects of nicotine treatment on neurodegeneration and locomotor activity in α-Syn tg mice. Twelve-month-old PDGFβ α-Syn-tg and non-tg mice were treated with nicotine (0.1 mg/kg intraperitoneal [IP]) or saline (= vehicle) twice daily for 2 weeks. Brain sections were immunohistochemically evaluated for neurodegeneration with the neuronal marker NeuN **(A, B)** and the dopaminergic marker anti-TH **(C, D)**. **(A)** Representative photomicrographs and **(B)** quantitation of the number of neurons in the neocortex, hippocampus, and striatum. While the number of NeuN-positive cells was unchanged in the neocortex and striatum, saline-treated α-Syn tg mice had significantly fewer neurons in the hippocampus than saline-treated non-tg mice. Nicotine treatment normalized the number of NeuN-positive cells in the hippocampus of α-Syn tg mice relative to saline-treated α-Syn tg mice. *N* = 8/group. Scale bar = 50 μm.^*^p < 0.0001, ^#^*p* < 0.001. **(C)** Representative photomicrographs and **(D)** quantitation of TH immunoreactivity in the substantia nigra (s. nigra), hippocampus, and striatum. While TH immunoreactivity was unchanged in the hippocampus and substantia nigra, saline-treated α-Syn tg mice exhibited significantly less immunoreactivity in the striatum than saline-treated non-tg mice. Nicotine treatment normalized TH immunoreactivity in the striatum of α-Syn tg mice relative to saline-treated α-Syn tg mice. *N* = 8/group. Scale bar = 50 μm. ^*^p < 0.05, ^#^*p* < 0.01. **(E)** The effect of nicotine treatment on locomotor activity was assessed using the rotarod test. Saline-treated α-Syn tg mice spent significantly less time on the rotarod than saline-treated non-tg mice. Nicotine-treated α-Syn tg mice remained on the rotarod for significantly longer than saline-treated α-Syn tg mice ^*^p < 0.05, *N* = 8 mice/group. In panels **(B, D)**, dots represent individual values and bars denote the mean ± standard error of the mean.

The original article has been updated.

