# Supplementary material for: Nicotine-mediated effects in neuronal and mouse models of synucleinopathy
Source: Front Neurosci. 2023 Aug 31;17:1239009. doi: 10.3389/fnins.2023.1239009 (PMC10501483; doi:10.3389/fnins.2023.1239009)
Supplement: Supplementary file 1 [file Data_Sheet_1.docx]

Supplementary Material


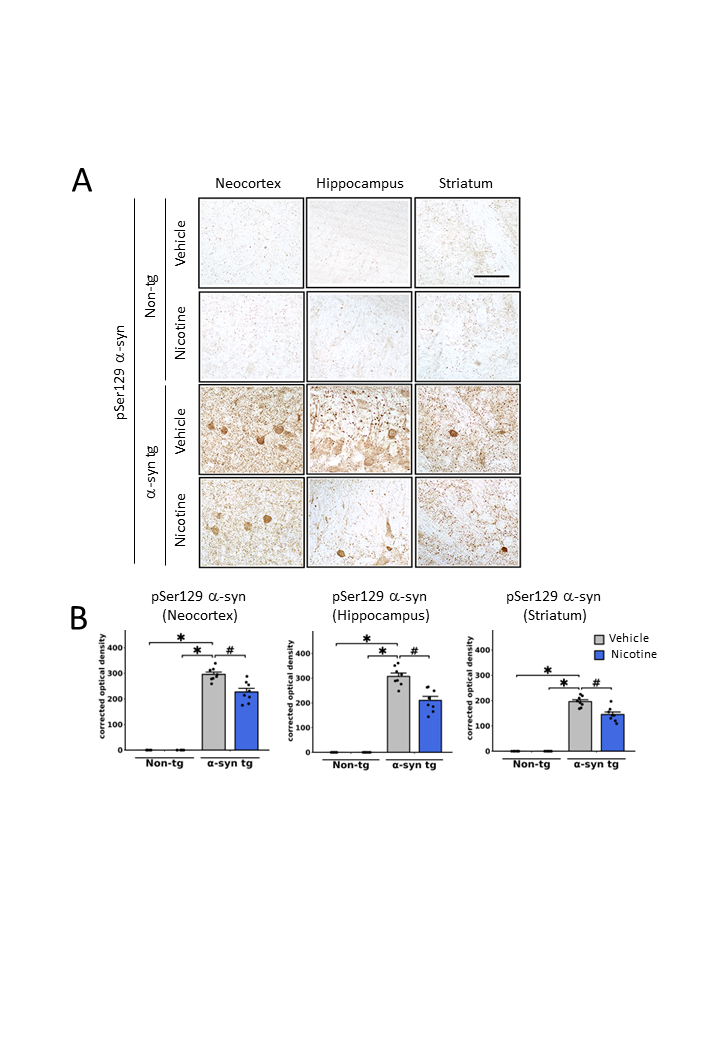


**Supplementary Figure 1**. Immunohistochemical evaluation of the effect of nicotine treatment on pS129 α-syn immunoreactivity in α-syn tg mice. Brain sections from 12-month-old PDGFβ α-syn mice and non-tg mice that were treated with nicotine (0.1 mg/kg intraperitoneal [IP]) or saline twice daily for 2 weeks and immunohistochemically analyzed for phosphorylated serine 129 (pSer129) α-syn immunoreactivity. **(A)** Representative photomicrographs and **(B)** quantitation of pSer129 α-syn immunoreactivity in the neocortex, hippocampus, and striatum. Saline-treated α-syn tg mice had a significant increase in the number of pSer129 α-syn inclusions in all three brain regions relative to saline-treated non-tg mice. Nicotine treatment significantly reduced the formation of pSer129 α-syn inclusions in the neocortex, hippocampus, and striatum relative to saline-treated α-syn tg mice. In **(A),** scale bar for all subpanels = 50 µm. In **(B)**, N = 8 mice/group, dots represent individual values and bars denote mean ± standard error of the mean. *P < 0.0001, ♯P < 0.0001.

**
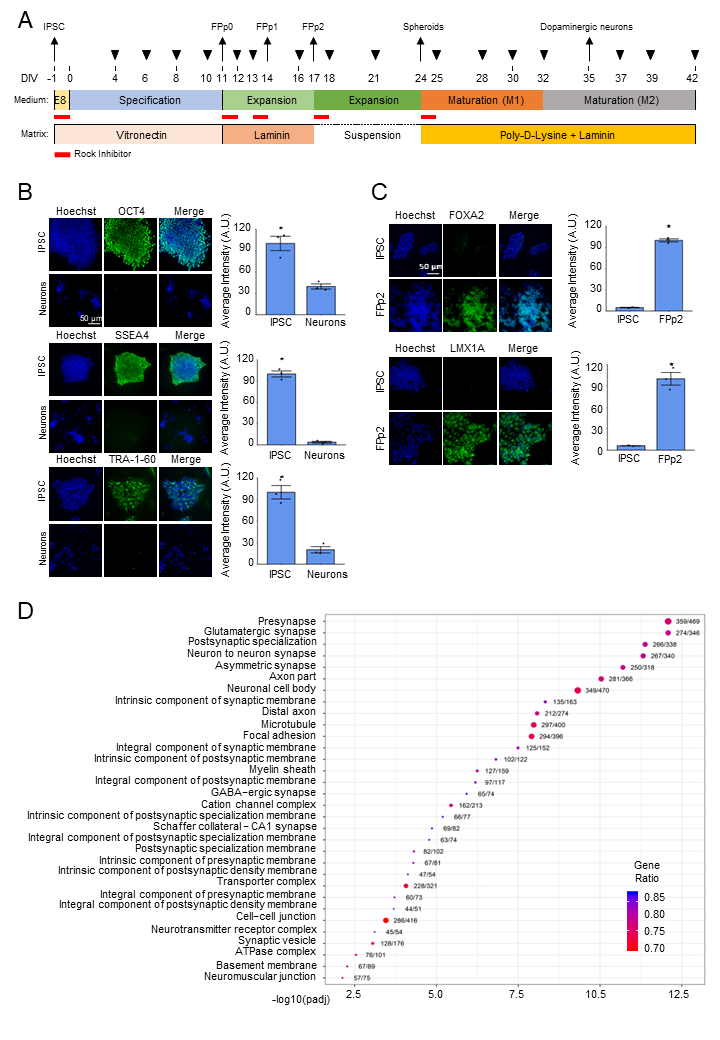
**

**Supplementary Figure 2.** Characterization of iPSCs, floor-plate progenitor cells, and differentiated dopaminergic neurons. **(A)** Outline of the protocol used for differentiation of iPSCs into dopaminergic neurons. iPSCs plated on vitronectin-coated plates are first induced in *floor-plate specification medium* for 10 days into midbrain-specified floor-plate progenitor (FPp) cells. Next, FPp cells are expanded on laminin-coated plates over 7 days in *floor-plate cell expansion medium* into FPp1 cells and, subsequently, to FPp2 cells, which are then banked in liquid nitrogen or directly expanded in suspension for 7 days to form spheres. Finally, the spheres are triturated and differentiated into mature dopaminergic neurons in *dopaminergic neuron maturation medium* (M1) and cultured on poly-D-lysine–laminin double-coated plates over the next 7 days. Mature neurons are further maintained in *dopaminergic neuron maturation medium* (M2), which contains B27. The entire workflow takes 35–42 days. E8 denotes essential 8 medium, and arrowheads indicate medium changes. **(B-C)** Immunostaining and automated quantification of total cellular intensity were performed to assess the expression of the stem cell markers octamer-binding transcription factor 4 (OCT4), SSEA-4, and TRA-1-60 in iPSCs versus differentiated dopaminergic neurons as negative control, as well as the expression of the FPp cell markers FOXA2 and LMX1A in floor-plate cells versus iPSCs as negative control. In **B–C**, N = 3 independent experiments, and bar plots represent mean ± standard deviation. *P < 0.05. **(D)** Enrichment analysis of the significantly altered genes (adjusted P < 0.05) in iPSCs versus neurons against “cellular component” GO terms. The *x*-axis corresponds to the enrichment false discovery rate (FDR)-adjusted P*-*value per GO term. The dot size is proportional to the number of genes differentially expressed in the GO term (left number near the dot). The right number near the dot corresponds to the total number of genes in the GO term and the color scale to the ratio of these two numbers.


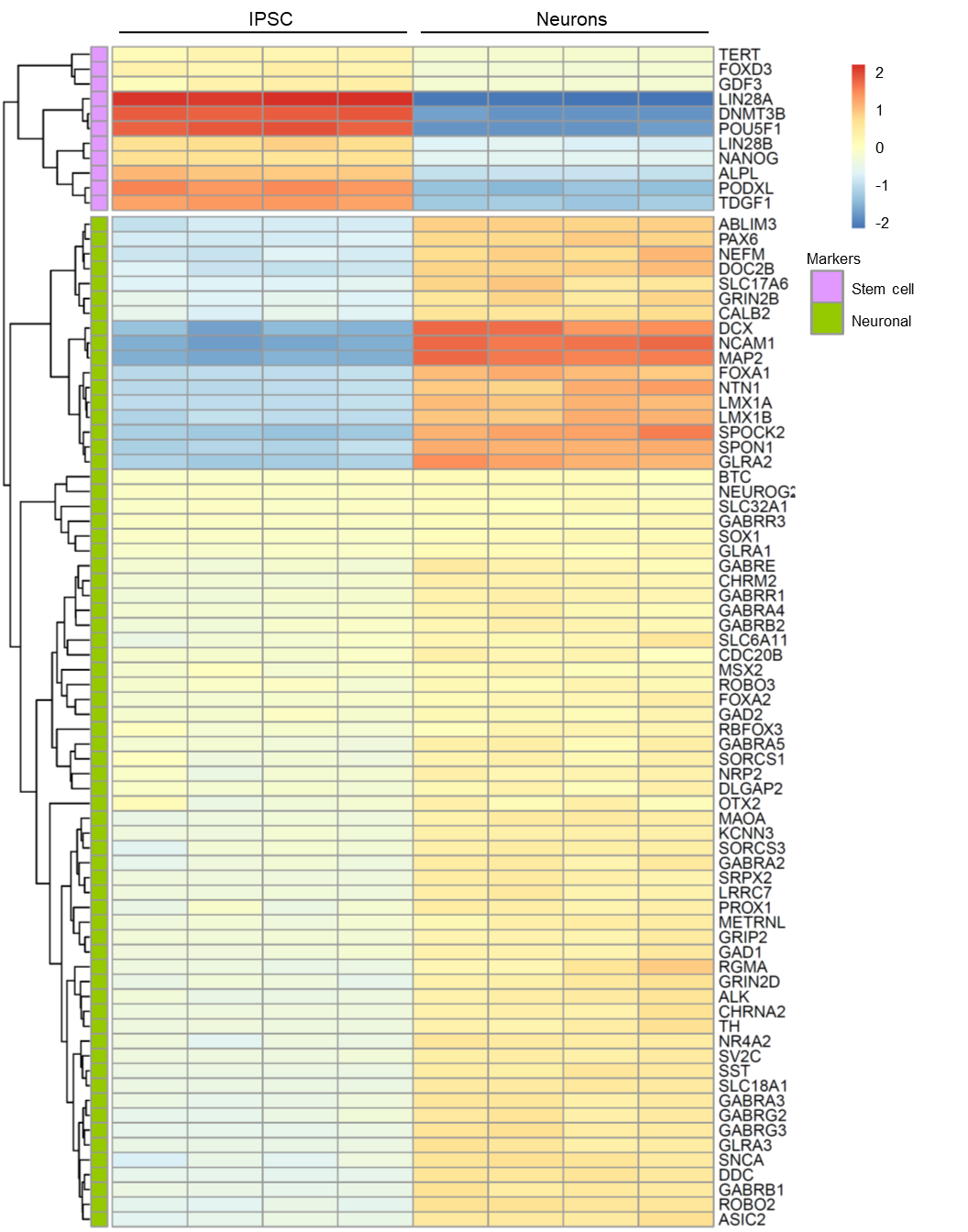


**Supplementary Figure 3.** Heatmap representing the expression levels of a panel of selected neuronal and stem cell markers in iPSCs before and after neuronal differentiation. RNA sequencing analysis shows that the expression of a wide range of mature and immature neuronal and dopaminergic markers is induced in neurons at 35 DIV relative to iPSCs. In contrast, the expression of a panel of stem cell markers is inhibited in neurons. The color scale indicates differences in gene expression in one replicate compared to the average gene expression across all replicates in both groups. The clustering is based on the Euclidean distance between the samples (for columns) or between the selected genes (for rows). n = 4 biological replicates and P < 0.05.


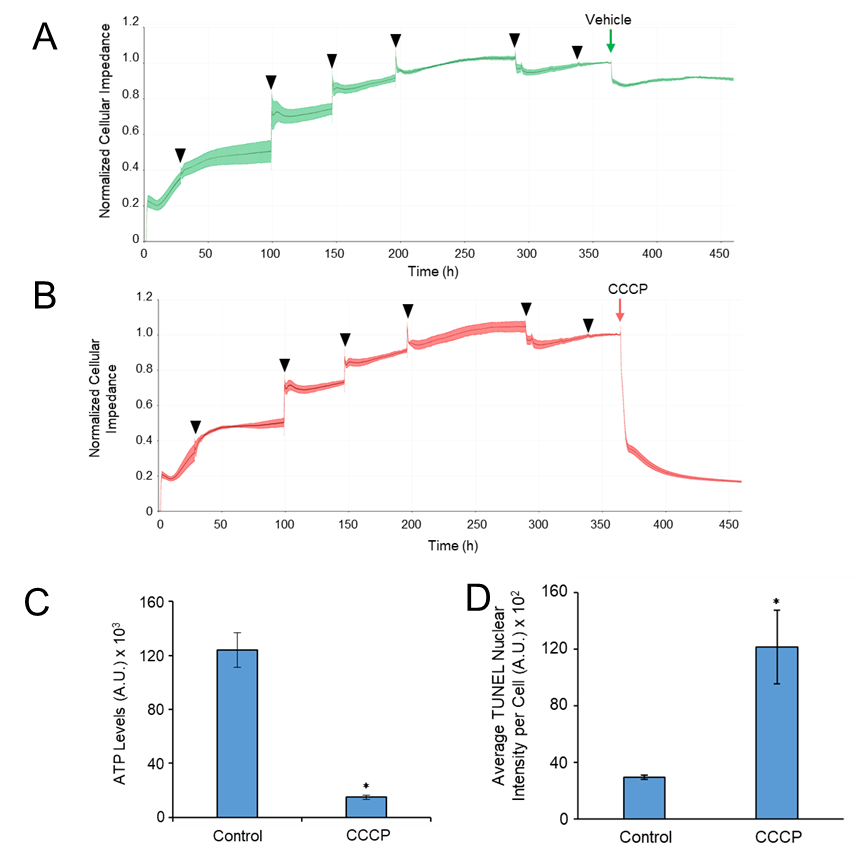


**Supplementary Figure 4.** Parallel confirmation of neuronal toxicity detected using the xCELLigence^™^ Real-Time Cell Analyzer (RTCA) using ATP and TUNEL assays. **(A-B)** Impedance of iPSC-derived neurons measured using RTCA following treatment with vehicle **(A)** or a toxic dose of CCCP **(B)** are shown as a function of time. Plots represent mean ± standard deviation. Arrowheads in **(A-B)** denote a change of impedance measurement due to medium changes. **(C-D)** Bar plots (mean ± standard deviation; *P < 0.05) correspond to the quantification of ATP content **(C)** and TUNEL reactivity **(D)** measured at the end of the RTCA recording. The decreased ATP content and increased apoptosis detected by the TUNEL assay confirm that the drop of impedance measured using RTCA after CCCP treatment is associated with increased cell death. N = 3 independent experiments. Abbreviations: A.U., arbitrary units; CCCP, carbonyl cyanide *m*-chlorophenyl hydrazine.


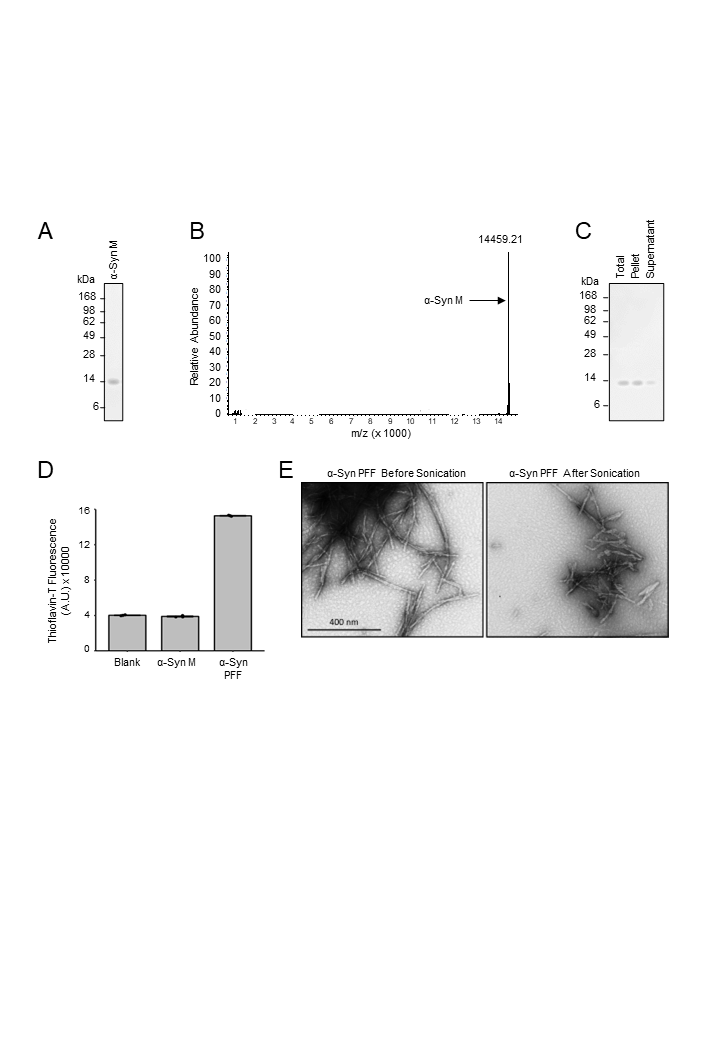


**Supplementary Figure 5.** Characterization of α-Syn monomers (α-Syn M) and preformed fibrils (PFF). **(A-B)** α-Syn M appear as a single monomeric band on SDS-PAGE stained by SYPRO-Ruby **(A),** and at the expected mass of ~14 kDa which was confirmed by ESI–MS analysis **(B).** α-Syn PFF generated by incubating α-Syn M for 5 days at 37℃ with shaking were characterized by SDS-PAGE **(C)**, which showed that α-Syn PFF sediment upon centrifugation (present mostly in the pellet rather than the supernatant fractions). **(D)** Assessment of Thioflavin-T binding (mean ± standard deviation) indicates that the α-Syn PFF exhibit β-sheet amyloid structure. “Blank” refers to vehicle solution, which is included as a control for background fluorescence. **(E)** Analysis of α-Syn PFF by transmission electron microscopy (TEM) validates their fibrillar nature and shows that sonication generates shorter fibrillar fragments. Abbreviation: A.U., arbitrary units.


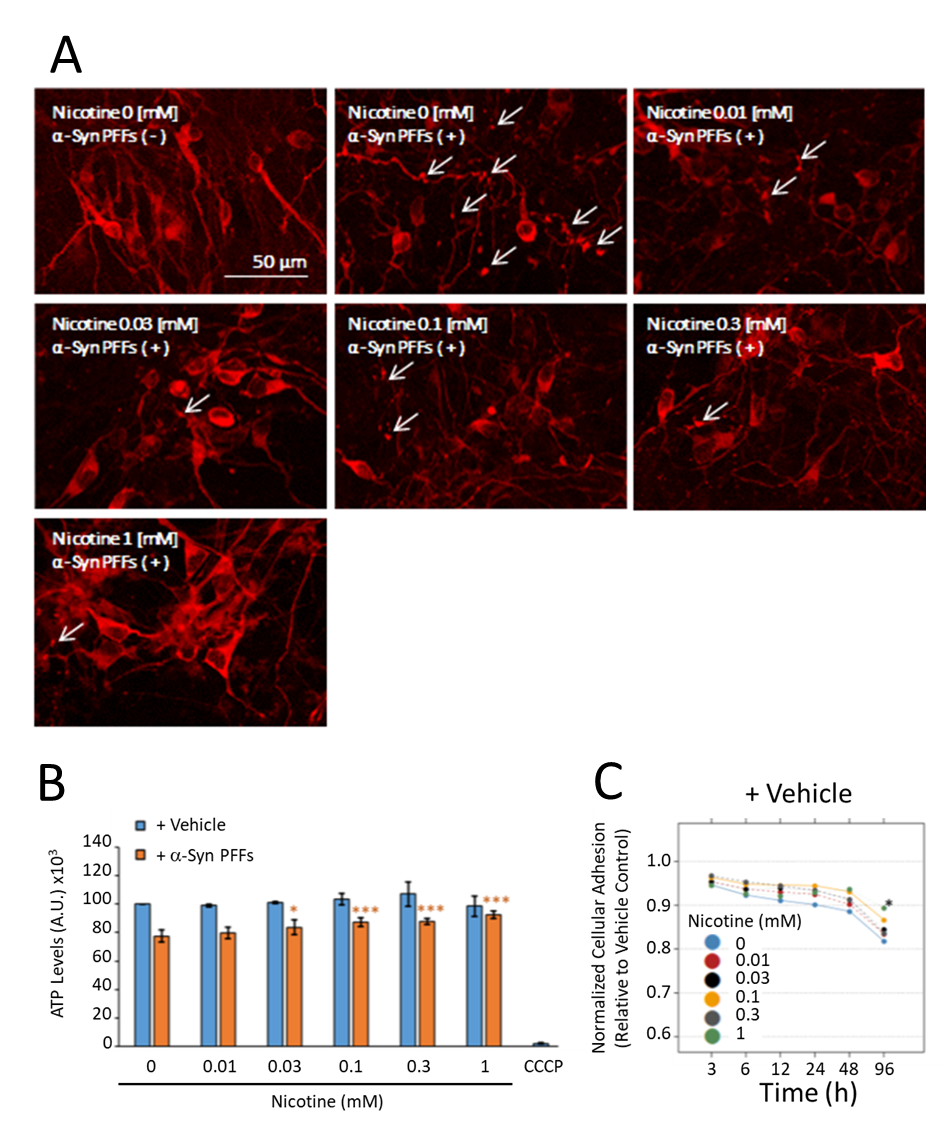


**Supplementary Figure 6.** Assessment of nicotine pretreatment effects on morphology, ATP levels, and adhesion of iPSC-derived neurons treated with α-Syn PFF. **(A)** Immunostaining of mature iPSC-derived neurons pretreated for 1 h with nicotine (0.03–1 mM) and then cotreated with α-Syn PFF or vehicle control for 96 h using antibodies against tyrosine hydroxylase shows that nicotine pretreatment attenuates the appearance of enlarged dystrophic neurites (white arrows). α-Syn PFF (+) indicates treatment with α-Syn PFF, and α-Syn PFF ( - ) indicates treatment with vehicle control. **(B)** Quantification of cellular ATP levels in mature iPSC-derived neurons treated as in **(A)** shows that nicotine significantly attenuates the observed drop in ATP levels due to α-Syn PFF treatment. Bar plots represent mean ± standard deviation (*P < 0.05, ***P < 0.001 compared to nicotine 0 mM) of N = 3 independent experiments. Abbreviations: A.U., arbitrary units; CCCP, carbonyl cyanide *m*-chlorophenyl hydrazine. **(C)** Real-time assessment of mature iPSC-derived neurons treated for 96 h with increasing doses of nicotine alone (0.01–1 mM) using the xCELLigence^™^ RTCA system shows that prolonged nicotine treatment per se reduces adhesion at 96-h post-treatment. N = 3 independent experiments, *P < 0.05.


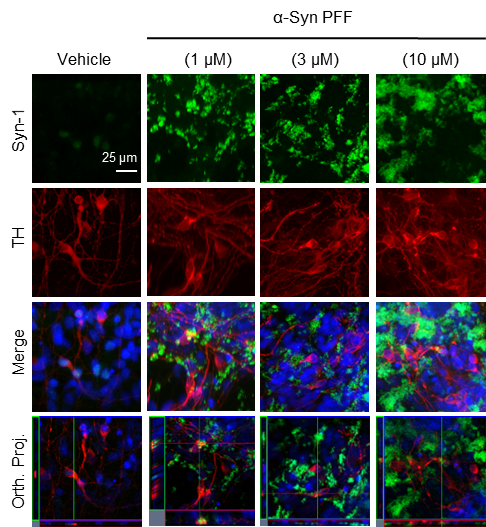


**Supplementary Figure 7.** Assessment of α-Syn PFF internalization in differentiated iPSC-derived dopaminergic neurons. Representative high-magnification confocal images of individual iPSC-derived dopaminergic neurons co-stained with α-Syn (green) and tyrosine hydroxylase (TH) (red) antibodies after 4 days of treatment with increasing doses of α-Syn PFF. Orthogonal projections (Orth. Pro.) of z-stacks establish the intracellular localization of PFF. Nuclear staining with Hoechst is in blue.


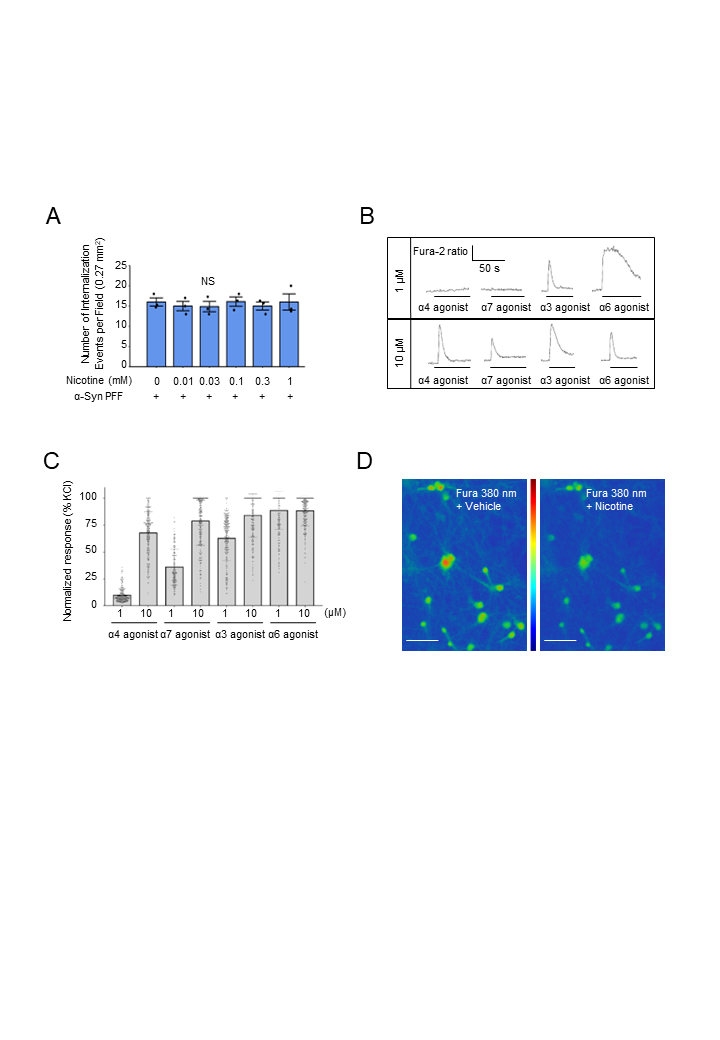


**Supplementary Figure 8. (A)** Quantification of internalization events by immunocytochemistry using the total α-Syn (Syn-1) antibody shows that nicotine pretreatment did not affect the number of dopaminergic neurons with internalized α-Syn PFF per field (0.27 mm^2^). N = 3 independent experiments. Dots represent individual values and bars denote the mean ± standard deviation. Abbreviation: NS, not statistically significant. **(B)** Fura-2 traces from iPSC-derived neurons in response to different specific agonists of nAChR tested at 1 mM or 10 mM. **(C)** Scatter plot of Fura-2 ratio peak calcium changes normalized to 10 mM KCl (n = 144-256 cells). Agonists used for α4: AZD1446; for α7: PNU282987; for α3: NS3861, for α6 and α4: 5-Iodo-A-85380. **(D)** Dopaminergic neurons displaying Fura-2AM fluorescence observed at 380 nm (unbound Fura) in color encoded map before (left panel) and after (right panel) nicotine-induced calcium intracellular increase.


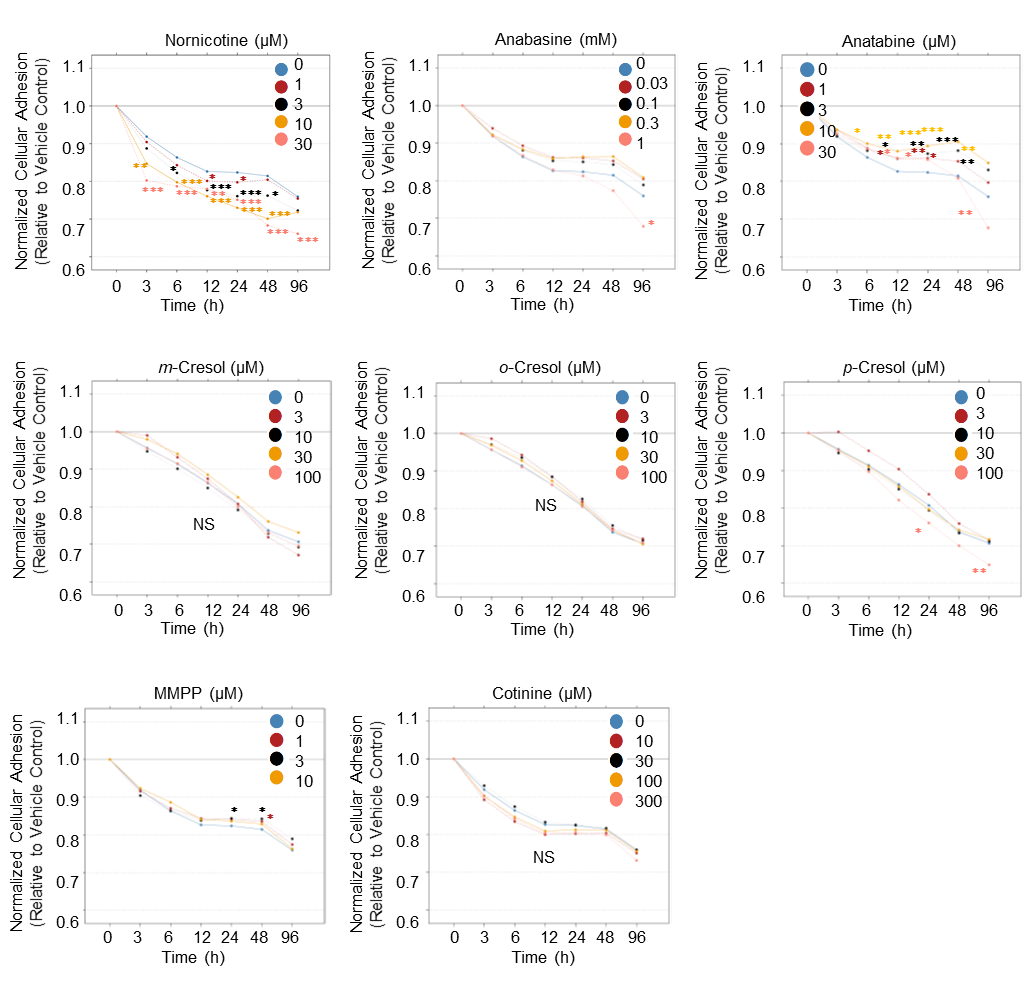


**Supplementary Figure 9.** Effect of pretreatment with different tobacco compounds on α-Syn PFF-provoked degeneration of iPSC-derived neurons. Mature iPSC-derived dopaminergic neurons were pretreated for 1 h with increasing doses of different tobacco compounds (nornicotine; anabasine; anatabine; MMPP; cotinine; *m*-, *o*-, and *p*-cresols; and PMI225), which was followed by 4 days of co-exposure to α-Syn PFF (3 µM). RTCA measurement of cellular adhesion shows a time-dependent decrease in all conditions, reflecting the α-Syn PFF-induced toxicity and the absence of protective effects of pretreatment with these compounds. Only anatabine pretreatment attenuates α-Syn PFF-provoked toxicity, mostly at 3 and 10 µM, and high doses of nornicotine and anabasine cause cumulative toxic effects with α-Syn PFF, indicated by a faster decrease in cellular adhesion. N = 3 independent experiments. *P < 0.05, **P < 0.01, ***P < 0.001. Abbreviations: iPSCs, induced pluripotent stem cells; NS, not statistically significant; PFF, preformed fibrils; RTCA, real-time cell analysis.

**Supplementary Table 1. Antibodies used in this study.**

| **Antibody** | **Catalog #** | **Supplier** | **Dilution** | |
| --- | --- | --- | --- | --- |
|  |  |  | **IHC** | **ICC** |
| MAP2 | 188006 | Synaptic Systems (Göttingen, Germany) | NA | 1:500 |
| Tyrosine hydroxylase | ab112 | Abcam | NA | 1:400 |
| Tyrosine hydroxylase | AB152 | Millipore  (Burlington, MA, USA) | 1:1,000 | NA |
| OCT4 | ab19857 | Abcam | NA | 1:300 |
| SSEA-4 | ab16287 | Abcam | NA | 1:100 |
| TRA-1-60 | ab16288 | Abcam | NA | 1:100 |
| FOXA2 | 701698 | Invitrogen  (Carlsbad, CA, USA) | NA | 1:100 |
| LMX1A | ab31006 | Abcam | NA | 1:200 |
| Iba1 | 019-19741 | FUJIFILM Wako Pure Chemical Corp.  (Osaka, Japan) | 1:1,000 | NA |
| α-Synuclein (Syn-1) | 610787 | BD Biosciences  (Franklin Lakes, NJ, USA) | 1:250 | 1:1,000 |
| pSer129 α-Synuclein | 010-26481 | Wako | 1:200 | NA |
| NeuN | MAB377 | Millipore | 1:1,000 | NA |
| GFAP | AB5804 | Millipore | 1:1,000 | NA |
| PSD95 | ab13552 | Abcam | NA | 1:100 |
| Synaptophysin | ab32127 | Abcam | NA | 1:100 |
| β3-Tubulin | T8660 | Sigma | NA | 1:1,000 |
| GIRK2 | ab219190 | Abcam | NA | 1:100 |

*Abbreviations: ICC, immunocytochemistry; IHC, immunohistochemistry; NA, not applicable.

**Supplementary Table 2. Tobacco compounds tested in this study.**

| **Full name** | **Short name** | **CAS** | **Supplier** | **Catalog #** |
| --- | --- | --- | --- | --- |
| (*S*)-Nicotine | Nicotine | 54-11-5 | Sigma-Aldrich  (St. Louis, MO, USA) | N3876 |
| (*R*,*S*)-Anatabine | Anatabine | 2743-90-0 | Indena  (Milan, Italy) | Lav 1056/17/D |
| (*R*,*S*)-Anabasine | Anabasine | 13078-04-1 | Sigma-Aldrich | 294599 |
| (*S*)-Cotinine | Cotinine | 486-56-6 | Sigma-Aldrich | C5923 |
| 1-Methyl-2-(1-methylpyrrolidin-3-yl)pyrrolidine | MMPP | 6602-17-1 | WuXi AppTec (Wuhan, China) | Not Available |
| (*S*)-*N*-Decanoyl-nornicotine | Nornicotine | 117642-91-8 | WuXi AppTec | Not Available |
| *m*-Cresol | *m*-Cresol | 108-39-4 | Sigma-Aldrich | W353000 |
| *p*-Cresol | *p*-Cresol | 106-44-5 | Sigma-Aldrich | W233706 |
| *o*-Cresol | *o*-Cresol | 95-48-7 | Sigma-Aldrich | W348007 |

**Supplementary Table 3. Compounds used for functional analysis.**

| **Full name** | **Short name** | **CAS** | **Supplier** | **Known effects/receptors specificities** | **EC_50_**  **(in mM)** |
| --- | --- | --- | --- | --- | --- |
| Acetylcholine | - | 60-31-1 | Sigma-Aldrich  (St. Louis, MO, USA) | nAChR agonist | ND |
| 3-(5-chloro-2-furoyl)-3,7-diazabicyclo(3.3.0)octane | AZD1446 | 1025007-04-8 | Key Organics (Camelford, UK) | α4 nAChR agonist | 7.9 ± 3.6 in α4 β2 nAChR; inactive in α7 and α3 |
| 3-(3-Bromo-2-thienyl)-8-methyl-8-azabicyclo[3.2.1]oct-2-ene | NS3861 | 216853-59-7 | Sigma-Aldrich | α3 nAChR agonist | 0.13 ± 0.02 in α3β4 nAChR; inactive in α7 and α4 |
| DMAB-anabaseine | - | 154149-38-9 | Tocris  (Bristol, UK) | Antagonist or partial agonist of nAChR | ND |
| Methyllycaconitine | MLA | 21019-30-7 | Sigma-Aldrich | nAChR antagonist | ND |
| 1,3-Dihydro-1-(3-*exo*)-9-methyl-9-azabicyclo[3.3.1]non-3-yl]-2*H*-indol-2-one | SR16584 | 1150153-86-8 | Cayman Chemical Company (Ann Arbor, MI, USA) | Selective α3β4 nAChR antagonist | ND |
| *N*-(3*R*)-1-Azabicyclo[2.2.2]oct-3-yl-4-chlorobenzamide | PNU282987 | 711085-63-1 | Cayman Chemical Company | α7 nAChR agonist | 3.4 ± 0.4 in α7Ric3 nAChR; inactive in α4 and α3 |
| 3-((2S)-2-Azetidinylmethoxy)-5-iodo-pyridine dihydrochloride hydrate | 5-Iodo A85380 dihydrochloride | 1217837-17-6 | Abcam  (Cambridge, UK) | α6β2 and α4β2 nAChR agonist | 0.01 in α6β2 nAChR;  0.03 in α4β2 nAChR; inactive in α7 and α3 |

*Note: The half-maximal effective concentrations (EC_50_) (for AZD1446, PNU282987, and NS3861) were derived from fitting the Hill equations of the concentration–response curves determined by using the whole-cell mode of the patch-clamp technique at a holding potential of −70 mV in CHO cells stably expressing human α4β2, human α7, and human α3β4 nAChR. The EC_50_ values of 5-Iodo-A-85380 were obtained from the literature (Mogg, A.J., Jones, F.A., Pullar, I.A., Sharples, C.G.V., Wonnacott, S., 2004, Functional responses and subunit composition of presynaptic nicotinic receptor subtypes explored using the novel agonist 5-iodo-A-85380. Neuropharmacology.47(6):848-59. doi: 10.1016/j.neuropharm.2004.06.013). Abbreviation: ND, not determined.
